# Targeting thymidine phosphorylase alleviates resistance to dendritic cell immunotherapy in colorectal cancer and promotes antitumor immunity

**DOI:** 10.3389/fimmu.2022.988071

**Published:** 2022-08-24

**Authors:** Ankush Paladhi, Samrat Daripa, Indrani Mondal, Sumit Kumar Hira

**Affiliations:** ^1^ Cellular Immunology Laboratory, Department of Zoology, The University of Burdwan, Purba Bardhaman, India; ^2^ Department of Hematology, Nil Ratan Sircar (NRS) Medical College and Hospital, Kolkata, India

**Keywords:** colorectal cancer, thymidine phosphorylase (TP), tumor immune microenvironment (TIM), dendritic cell (DC) vaccine, T-cell exhaustion, immunogenic cell death (ICD)

## Abstract

T-cell exhaustion plays a pivotal role in the resistance of microsatellite-stable colorectal cancer (CRC) to immunotherapy. Identifying and targeting T-cell exhaustion-activating mechanisms is a promising strategy to augment the effects of immunotherapy. Here, we found that thymidine phosphorylase (TYMP) plays a decisive role in inducing systemic T-cell exhaustion and abrogating the efficacy of dendritic cell (DC) therapy in a CRC model. Targeting TYMP with tipiracil hydrochloride (TPI) induces immunological cell death (ICD). The combined effects of TPI and imiquimod-activated DCs turn CT26 tumors into immunologically ‘hot’ tumors by inducing ICD *in vivo*. High-dimensional cytometry analysis revealed T-cell and IFN-γ dependency on the therapeutic outcome. In addition, chemoimmunotherapy converts intratumoral Treg cells into Th1 effector cells and eliminates tumor-associated macrophages, resulting in higher cytotoxic T lymphocyte infiltration and activation. This effect is also associated with the downregulation of PD-L1 expression in tumors, leading to the prevention of T-cell exhaustion. Thus, cooperative and cognitive interactions between dendritic cells and immunogenic cell death induced by therapy with TPI promote the immune response and tumoricidal activities against microsatellite stable colorectal cancer. Our results support TYMP targeting to improve the effects of DC immunotherapy and outcomes in CRC.

## Introduction

Chemotherapy regimens, including 5-fluorouracil (5Fu), oxaliplatin, irinotecan, and leucovorin acid, form the backbone of colorectal cancer (CRC) treatment (1). The addition of monoclonal antibodies (mAbs) (2) or immune checkpoints (3) has largely improved clinical outcomes in several CRC types and has induced a high rate of clinical response in mismatch repair-deficient and high-level microsatellite instability (dMMR-MSI-H) subgroups (~15% of patients with metastatic CRC) (4). However, immunotherapies involving current immune checkpoint blockers (ICBs) remain unresponsive in cases of mismatch repair proficiency and have low levels of microsatellite instability (pMMR-MSI-L) in subgroups (~85% of patients with metastatic CRC) (5). In addition to ICBs and targeted mAbs, cell therapies involving dendritic cells (DCs) remain an exciting immunotherapy arm (6, 7). Despite the promise of these immunologically targeted approaches, the clinical response to targeted immunotherapies or DC vaccines has been largely disappointing (8, 9). This is because of the reduced expression of neoantigens and largely excludes functional cytotoxic T lymphocytes (CTLs) from the tumor microenvironment (TME), although they may contain small numbers of exhausted CTLs (4, 10). The poor tumor penetration of mAbs and the immunosuppressive TME limit the immunotherapeutic efficacy of ICBs and cell therapies for CRC (11, 12). Therefore, relieving immunosuppression in the TME is one strategy to enhance immunotherapeutic efficacy against CRC.

Thymidine phosphorylase (TYMP), a rate-limiting enzyme in thymidine catabolism (13) and similar to platelet-derived endothelial cell growth factor (PD-ECGF), plays a key role in 5FU pharmacokinetics (1), the inflammatory response, neoangiogenesis, and apoptosis (14). TYMP expression in various solid tumors, such as head–neck, breast, lung, oral squamous carcinoma, esophageal, gastric, bladder, prostate, ovarian, and cervical cancers, is higher than that in adjacent noncancerous tissues (15), including colorectal carcinomas (16). In many cases, poor prognosis is associated with TYMP-positive versus TYMP-negative colons and differentiated gastric carcinomas (17, 18). TYMP has several tumor-promoting functions and is positively correlated with angiogenic factors such as vascular endothelial growth factor (VEGF) and microvessel density. Several studies have indicated that TYMP inhibits Fas-induced apoptosis by interfering with the release of cytochrome c and caspase-3 cleavage. Increased TYMP expression suppresses the immune response via increased secretion of IL-10 in the TME, which inhibits the effector functions of DCs and the Th1 cytokine profile (19). TYMP-targeted therapy using small-molecule TYMP inhibitors (i.e., trifluridine, tipiracil, or TAS-102) has been shown to cause tumor regression in a large number of preclinical gastrointestinal tumor models (20–23) and is under evaluation as monotherapy (NCT03974594) and in combination with other therapeutic regimens in a large number of clinical trials (24–26). While these studies are insightful, the mechanisms mediating the therapeutic response in CRC for anti-TYMP alone or in combination with cell therapies, such as adoptive DC vaccines, remain to be elucidated. Specifically, the effect of TYMP silencing on the CRC TME, including the status of exhausted CTLs, is poorly understood. Moreover, it is unclear whether TYMP-targeted therapy depletes or destabilizes exhausted T cells and regulatory T cells (Tregs) in the TME and how this therapy cooperates with DC therapy to improve prognosis.

Here, we tested the immunomodulatory effects of the TYMP inhibitor tipiracil hydrochloride (TPI), alone and in combination with a TLR7 agonist (imiquimod), activated whole tumor lysate-pulsed DCs (IMQDC-Ag), both in vitro and in vivo. We found that TYMP plays a decisive role in inducing systemic T-cell exhaustion and abrogating the efficacy of DC therapy in a microsatellite stable CRC model. For the first time, we report that chemoimmunotherapy abolizes immunotherapy resistance induced by TYMP. In addition, chemoimmunotherapy converts intratumoral Treg cells into Th1 effector cells and eliminates tumor-associated macrophages, resulting in higher cytotoxic T-cell infiltration and activation. This effect is also associated with the downregulation of PD-L1 expression in tumor cells, leading to the prevention of T-cell exhaustion.

## Materials and methods

### Ethics statement

Animal studies were conducted with the approval of the Institutional Dissection Monitoring Committee (DMC) at the University of Burdwan (BU-DMC/2016/01/02).

### Materials and reagents

Information about the materials (medium, pharmacological inhibitors, plasticware, etc.), reagents (i.e., antibodies, chemicals and recombinant proteins, ELISA kits, critical commercial assay kits), and resources (software) are summarized in **Supplementary Tables 1-8** included in the supplementary information file.

### Cells and animals

Details of the cell lines and mouse strains used were based on our previously described studies (6, 27), and details are given in the Supplementary Materials and Methods section.

### BMDC culture and stimulation

BMDCs were isolated from femurs of BALB/c mice as previously described (28). Details are provided in the Supplementary Materials and Methods section.

### In vitro antitumor efficacy and apoptosis detection

The antitumor efficacy of the thymidine phosphorylase inhibitor tipiracil hydrochloride (TPI) was evaluated, as previously described (6). To assess TPI-mediated apoptosis of CT-26 cells, Annexin-FITC/PI staining was performed as described previously (6). Details are provided in the Supplementary Materials and Methods section.

### DC functional assays

Mouse BMDCs or human peripheral blood DCs were cultured in 96-well plates with medium alone (naïve DCs) or in the presence of GM-CSF (1000 U/mL) (GM-CSF DCs), imiquimod (200 pg/mL) (IMQ DCs), or LPS (5 µg/mL) (LPS DCs). To assess DC-mediated growth inhibition (48 h), an MTT assay was performed, as described previously (28). Details are provided in the Supplementary Materials and Methods section.

### Transplantable CT-26 tumors

Tumor formation was induced by subcutaneous injection of 5×10^4^ CT-26 cells in 100 µL of serum-free RPMI into the hindquarters of BALB/c male mice (27). Mice were randomized into experimental groups when subcutaneous tumors reached 50 mm^3^. Ten days after tumor cell implantation, when the average tumor size reached 90 mm^3^, monitoring was initiated (day 0). The tumor volume was calculated as (length × width × height)/2.

### Antitumor efficacy against s.c. CT-26 tumors in Balb/c mice

Male BALB/c mice (6-8 weeks old, Charles River Laboratories, n=6) were injected subcutaneously into the right flank with CT-26 cells (5 × 10^4^ cells in 0.1 mL). Mice were randomized into groups, and tipiracil hydrochloride (TPI) treatment was initiated when the average tumor volume was ~80-100 mm3 on day 10. For the control group, vehicle (0.5% HPMC solution, 10 mL/kg) was orally administered. TPI at the indicated doses (50, 100, and 150 mg/kg/day) was orally administered twice a day, followed by one drug-free day for 4 days. After a gap of 6 days, TPI was again administered orally once a day, followed by one drug-free day for 8 days.

CT-26 tumor-bearing mice (n=6) were treated with eight intraperitoneal (i.p.) injections of whole CT-26 tumor lysate-pulsed DCs or imiquimod-activated CT-26 whole tumor-pulsed DCs (1×10^6^ cells/mouse) either alone or in addition to TPI (50 mg/kg body weight) by oral gavage for 22 days. Altogether, six TPI doses and eight DC doses were given at intervals of 24 hours. Therapeutic efficacy was determined by calculating the tumor burden for all mice over the course of the study using the area under the curve (AUC) of tumor volume over time. The adjusted AUC values for all mice in each group were analyzed using nonparametric analysis of variance (ANOVA on ranks). Furthermore, the incidence, magnitude, and durability of regression responses were evaluated. An animal with a complete regression response (six consecutive measurements) at the termination of the study was classified as a tumor-free survivor. Survival was analyzed using the Kaplan–Meier method. Groups of mice were compared using the log-rank test.

### Histology, immunohistochemistry and immunofluorescence

Unless otherwise specified, all histology, immunohistochemistry (IHC), immunofluorescence (IF) analysis and TUNEL assays were performed in formalin-fixed, paraffin-embedded tissue sections as described earlier (6, 29, 30). Details are provided in the Supplementary Materials and Methods section. The antibodies used for IHC and IF staining are listed in **Supplementary Table S4**.

### Western blot

Western blot analysis of the target proteins (listed in **Supplemental Table S5**) was performed as described previously (6, 28). Details are provided in the Supplementary Materials and Methods section.

### Measurement of ATP release

ATP release in untreated or TPI-treated CT-26 cells was measured using the commercial ATP Bioluminescence Assay Kit HS II (11699709001) from Roche, USA, according to the manufacturer’s instructions, as described previously (30).

### Tumor-infiltrating lymphocyte analysis for in vivo antitumor immune response

Tumor-infiltrating lymphocyte analysis was performed using flow cytometry as described previously (6). Details are provided in the Supplementary Materials and Methods section.

### Cytokine quantification

Cytokine quantification was performed according to the manufacturer’s instructions using a custom mouse cytokine multiplex assay panel (LEGENDplex™ MU Th1/Th2 Panel (8-plex), BioLegend, catalog no. 741053). The panel includes IL-5, IL-13, IL-2, IL-6, IL-10, IFN-γ, TNF-α, and IL-4, which are collectively secreted by Th1 and Th2 cells. Details are provided in the Supplementary Materials and Methods section.

### Statistics

All statistical analyses were performed using Origin2021b, SAS, R, or GraphPad Prism 9. One-way analysis of variance (ANOVA) and two-way ANOVA were used to compare continuous outcomes across multiple experimental groups. For all tests, P < 0.05 was considered significant (NS no significance; *P < 0.05, **P < 0.01, ***P < 0.001, and ****P < 0.0001). However, the sample size was not predetermined in this study. Unless otherwise noted, the samples were used as independent biological triplicates. For in vitro and in vivo studies, analysis of variance and t- and chi-squared tests were used to compare independent groups. Survival functions were estimated using the Kaplan–Meier method and compared using the log-rank test.

## Results

### High-dimensional immune profiling of CRC tumors reveals distinct immunotypes correlated with TYMP overexpression

**Figure 1:**
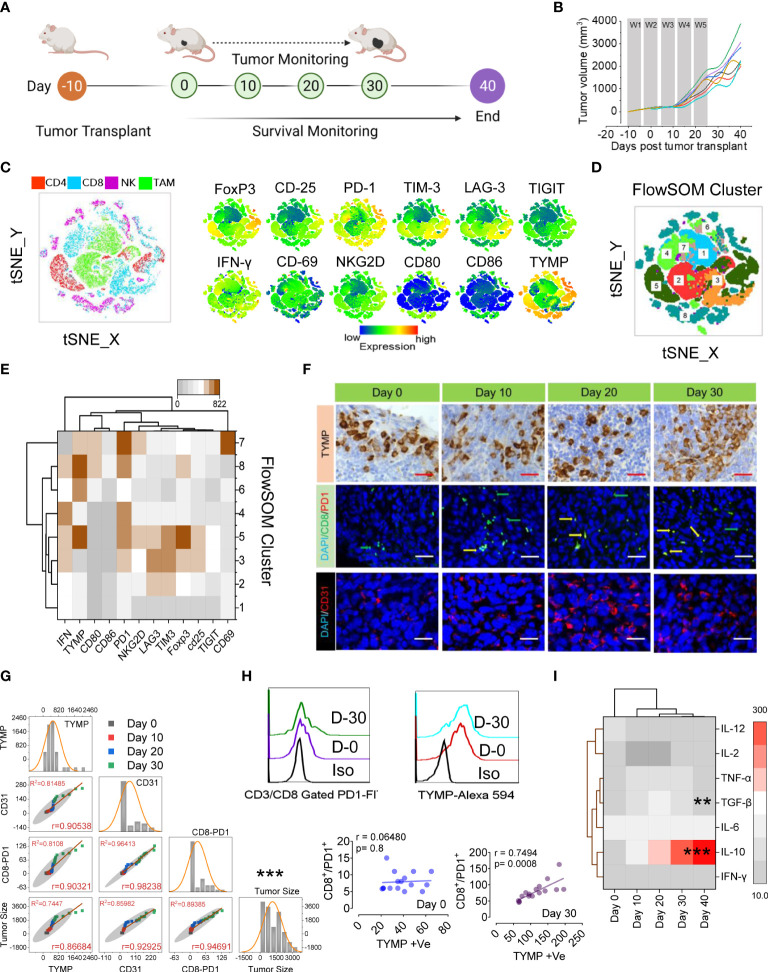
PD1^Hi^ T cells and increased Treg cells correlate with TYMP overexpression in colorectal carcinoma, which results in poor prognosis and survival. **(A)** Experimental schedule; after 10 days of s.c. inoculation with 5 × 10^4^ CT-26 cells, the tumors reached approximately 90 mm^3^, and tumor monitoring was initiated (noted as Day 0). **(B)** The tumor growth curves, **(C)** tSNE map of CD4^+^ T cells in TILs (red dots), CD8^+^ T cells (blue dots), NKG2D^+^ NK cells (pink dots) and CD11b^+^/Gr-1^lo^ TAM cells (green dots) of the polled CRC tumors (n = 6). tSNE maps showing the expression level and the distribution of each indicated marker. The color scale indicates the relative expression levels. **(D)** tSNE projection of CD8 T-cell clusters identified by FlowSOM clustering. **(E)** Mean fluorescence intensity (MFI) as indicated (column-scaled z scores) markers for selected clusters. The color scale indicates the relative expression levels. **(F)** IHC analysis of TYMP (top), double immunofluorescence staining for PD-1 and CD8 (middle) and immunofluorescence analysis of CD31 (bottom) in CRC tumors at the indicated time points. **(G)** Multivariate scatter plot matrix correlation analysis showing histograms, absolute correlations, and correlation coefficients (r) for the relationship between TYMP, CD31, and CD8-PD1 cells and the tumor growth rate over time. **(H)** Representative PD1 expression in CD3/CD8-gated TILs and TYMP expression in CRC tumors at days 0 and 30. Linear regression analysis with a linear mixed model showing the relationship between CD8^+^/PD1^+^ cells and TYMP expression in tumors on the indicated days. **(I)** Heatmap showing the normalized cytokine expression level in serum. The color scale indicates the relative expression levels. Significance was determined by an unpaired Wilcoxon test with Benjamini and Hochberg correction. Unless otherwise noted, the data are presented as independent biological triplicates. *In vivo* studies were repeated independently five times to confirm the results. *p < 0.05, **p < 0.01, ***p < 0.001.

Several clinical studies (31, 32) have indicated that TYMP expression is associated with time to progression in patients with metastatic colorectal cancer. To investigate the relationship between TYMP and the immune response against CT-26 tumors (pMMR-MSI-L and Kras-mutant) (33), we established subcutaneous (s.c.) CT-26 solid tumors to model advanced disease (27) and compared the TILs using high-dimensional flow cytometry. After 10 days of subcutaneous inoculation with 5×10^4^ CT-26 cells into the hindquarters of Balb/c male mice, the tumors reached approximately 90 mm^3^, and tumor monitoring was initiated (noted as day 0), as shown in **Figure 1A**. We found a noticeable increase in tumor volume between days 15 and 30, and all inoculated sites had large tumor nodules (**Figure 1B**). Tumors were excised at different time points (days 0, 10, 20, and 30), mononuclear cells were purified, and we first focused on the major lymphocyte populations among the TILs (**Figure 1C**). The number of CD3+ T cells decreased as the tumor grew (**Supplementary Figures S1C–D**). Examination of only CD3+ T cells revealed a preferential loss of CD8+ T cells compared with CD4+ T cells (**Figures S1C, D**). Among the CD8+ cells in tumors, approximately 84% expressed the PD-1+Tim-3+ phenotype that has been described for “exhausted” cells in mice with tumors. Among the CD4+ cells in the tumors, approximately 48% were CD25+, and among the latter, approximately 70% were FoxP3+ Treg cells (**Figure S1D**). Interestingly, mononuclear cells in tumors contained approximately 26% CD11b+Gr-1^hi^ cells (MDSC phenotype) and 19% CD11b+Gr-1^lo^ cells (TAM phenotype) (**Figures S1E, F**).

We next applied a multiparametric high-dimensional flow cytometric analysis to further investigate the TIL phenotype and functional state. **Figures 1C–E** compares the representative profiles of the 30-day tumors from the tumor-bearing mice. Along with Tregs (CD25+, FoxP3+), the expression of activation markers (IFN-γ, CD69, CD80, CD86, and NKG2D), inhibitory receptors (PD1, TIM3, LAG3, and TIGIT) and TYMP was assessed in TILs. To assess whether these markers might show the emergence of unique T cells infiltrating CRC tissue, we performed a t-distributed stochastic neighbor embedding (tSNE) algorithm that reduces all data to two dimensions (**Figures 1C, D**). To better characterize the CRC-associated clusters, tSNE analysis was compared with individual CD4+ (red), CD8+ (blue), NK (pink), and tumor-associated macrophages or TAM (green) subpopulations (**Figure 1C**). High expression of inhibitory receptors mostly contributed to the differential clustering of CD8+ TILs and FoxP3/CD25 in CD4+ TILs. A small population of IFN-γ-expressing CD8+ T-cell cells was identified among the clusters. Surprisingly, the high expression of TYMP mostly contributed to different CD4+ cells and TAMs (**Figure 1C**). To further define and quantify CD8+ T cells, we performed FlowSOM clustering (**Figure 1D**) and compared the expression of 12 CD8+ T-cell markers to identify each cluster (**Figure 1D**). This approach identified an increase in CD8+ T cells in several clusters, including clusters 5, 6, 8, and 7, in CT-26 tumors, reflecting high levels of TYMP and PD1 and differing expression of other activation markers and inhibitory receptors (**Figure 1E**). Cluster 7 expressed high levels of CD69, suggesting that they were tissue-resident cells within the tumor, although this marker may also indicate antigen stimulation (**Figure 1E** and **Figure S1D**). Collectively, these data show that CD8+ T cells coexpressing PD1 and TYMP preferentially accumulated in the tumor.

To investigate the potential correlations between TYMP and neoangiogenesis and CD8+/PD1+ presence in immune infiltrate CT-26 tumors, we performed immunohistochemical localization of TYMP, CD31, and CD8/PD1 in tumor tissue (**Figure 1F**) and performed multivariate correlation analysis (**Figures 1G, H**). The data showed a positive correlation between TYMP and T-cell exhaustion (r=0.7494, p=0.0008). Furthermore, we examined the anti- and proinflammatory cytokine profiles of CT-26 tumor-bearing mice to identify the humoral response against CT-26 tumors via cytokine generation. Our data suggest that serum IL-10 levels increase with tumor progression, whereas the level of TGF-β initially indicates that it inhibits epithelial growth, and the latter (day 40) appears to promote tumor progression (**Figure 1I**). This analysis revealed that the microenvironment of CT-26 colon tumors is highly immunosuppressive. Other relationships were also apparent, including correlations between TYMP overexpression and definite immunotypes or metrics of disease extremity in CT-26 tumors (**Figures 1G, H**).

### Increased TYMP expression in CRC tumors restrains adoptive dendritic cell therapy efficacy by inducing T-cell exhaustion

DCs have received appreciable attention as prospective targets for the development of anticancer vaccines (6, 28). Toward DC vaccines, designing ex vivo modification (activation and maturation) steps presents an important strategy for improving vaccine efficacy. Similar to several other DC-activating agents (i.e., IL-15), Toll-like receptor (TLR) agonists have attracted attention as potential therapeutic adjuvants. Although proof of principle for this concept has been proposed with agonists of several TLRs (3, 7, and 9), only one TLR7 agonist (imiquimod) has been approved for clinical use (34), although the clinical efficacy of this vaccine remains suboptimal. To test our hypothesis that increased TYMP expression in CRC tumors restrains adoptive dendritic cell therapy efficacy by inducing T-cell exhaustion, we used imiquimod-stimulated (IMQDC) bone marrow-derived DCs (BMDCs) pulsed with or without the whole CT-26 tumor antigen (Ag) as a therapeutic agent against CT-26 tumors in vivo and in vitro.

**Figure 2:**
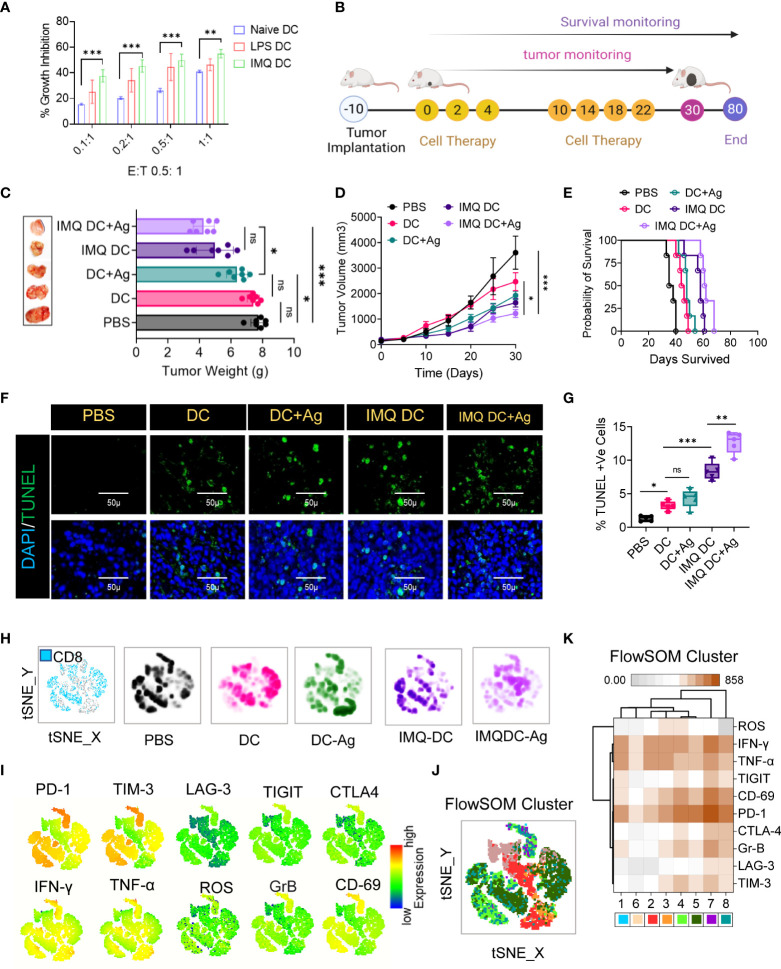
Robust exhausted T-cell populations in CRC mice restrain DC immunotherapy efficacy. **(A)** *In vitro* tumoricidal activity of naïve and imiquimod (200 pg/mL)-activated DCs was assessed by MTT assay. LPS (10 μg/ml) was used as a positive control. Treatment of CRC tumors: **(B)** Experimental schedule; after 10 days of s.c. inoculation with 5 × 10^4^ CT-26 cells when tumors reached approximately 90 mm^3^, the treatment was initiated (noted as Day 0) with PBS, DCs, IMQ-activated DCs or DCs pulsed with whole tumor antigen every 2 days for a total of 6 times. **(C)** The tumor weight (mean ± SD, n=6) of each indicated group and representative images of the tumors dissected on the indicated days. **(D)** Tumor growth curves (n=6). **(E)** Kaplan–Meier analysis of the mice; n = 6 mice. **(F)** TUNEL staining of the tumor sections dissected on day 30; green: TUNEL; blue: DAPI; scale bars: 50 μm; the experiment was repeated independently three times to confirm the results. **(G)** Boxes represent interquartile ranges (IQRs) of the percentage of TUNEL-positive cells. The experiment was performed in triplicate with four mice, and the results are presented as the mean ± SD. **(H)** Global tSNE projection of nonnaïve CD8^+^ T cells for all pooled (blue) and indicated treatment groups. **(I)** tSNE maps showing the expression level and the distribution of each indicated marker. The color scale indicates the relative expression levels. **(J)** tSNE projection of CD8^+^ T-cell clusters (colored differently) identified by FlowSOM clustering. **(K)** Mean fluorescence intensity (MFI) as indicated (column-scaled z scores) markers for selected clusters. The color scale indicates the relative expression levels. Statistical significance was analyzed by two-tailed Student’s t test **(C, D)** or by the log-rank (Mantel–Cox) test **(E)**. Unless otherwise noted, the data are presented as independent biological triplicates. *In vivo* studies were repeated independently five times to confirm the results. ns, not significant, *p < 0.05, **p < 0.01, and ***p < 0.001.

The in vitro growth of CT-26 cells was significantly inhibited in the presence of activated BMDCs. IMQ had a more pronounced effect than naïve DCs at all E:T ratios tested (**Figure 2A**). LPS-treated DCs were used as positive controls for DC activation. Next, we tested the effect of IMQDCs on CT-26 tumor-bearing mice. Similar to the schedule presented in **Figure 2B**, an ACT schedule was formulated. Naïve DC monotherapy exhibited no significant tumor inhibition ability in vivo, and IMQDC+Ag slowed tumor growth (weight and size) (**Figures 2C, D**) but regained growth momentum after the treatment stopped at day 22. Kaplan–Meier survival analysis showed that IMQDC+Ag treatment resulted in slightly higher (60 days) survival than BMDCs alone (all died at day 50) and untreated animals between days 35-40 (**Figure 2E**). The lack of antitumor capability of DC monotherapy is reflected in the continuous tumor growth and inefficacy in improving the survival of treated animals. Eight days after the last therapy, four mice from each group were sacrificed, and paraffin sections of excised tumors were analyzed by TUNEL staining (**Figure 2F**). Compared to the tumor sections treated with IMQDCs and DCs with or without Ag, the IMQDC+Ag-treated tumors contained pervasive apoptotic cells (**Figure 2G**). Tumor necrosis is often observed and a common pathological feature of growing tumors and is considered a morphological marker, with poor prognosis in a variety of cancer tumors. It has been documented that ischemic injury due to uncontrolled proliferation without efficient vasculature correlated with necroptosis of tumor cells leads to tumor necrosis and promotes tumor metastasis (35). The presence of tumor necrosis (% necrosis area) identified in untreated (30.9 ± 7.25, n=13) or DC monotherapy (16.71 ± 7.18, n=13) was significantly reduced in the IMQDC+Ag group (2.52 ± 1.39, n=13) (**Figures S2A, C**). Examination of the proliferation marker Ki-67 revealed that tumors from all treatment groups exhibited robust proliferation at the invasive margins (**Figures S2B, E**), except for the IMQDC+Ag group.

Considering that the treatment groups receiving DC immunotherapy have robust proliferation, it is important to understand the distinct characteristics of effector and exhausted CTLs among the TILs. To investigate the immunological characteristics of CD8+ TILs, we first compared the expression of immune checkpoint receptors (PD-1, Tim-3, LAG-3, TIGIT, and CTLA-4) and activation markers (IFN-γ, TNF-α, ROS, granzyme B or GrB, and CD69) (**Figure S3**). Combined and individual t-SNE analysis of paired CD8+ TILs from treatment groups (n=25, samples) revealed 22 different clusters (**Figure 2H, I**), which were further grouped into eight subpopulations by FlowSOM clustering based on the differential expression of checkpoint receptors and activation markers (**Figure 2J**). Among these eight distinct subpopulations, we found that the PD-1^high^ subpopulation exhibited the highest proportion in almost all treatment groups, as well as the highest proportion of the TIM3+ subpopulation (**Figure 2I**). FlowSOM clustering (**Figure 2J**) and comparison of the expression of 10 CD8+ T-cell markers in each identified cluster (**Figure 2K**), excluding cluster 6, in CT-26 tumors reflected high levels of PD1 and CD69 and differing expression of other activation markers and inhibitory receptors (**Figure 2K**). Observations revealed severe T-cell exhaustion features similar to those of the untreated group in the case of DC vaccination. It is poorly understood whether TYMP overexpression in tumor tissues contributes to cancer mortality by compromising systemic antitumor immunity and limiting cell therapy efficacy. Next, to assess the clinical significance of TYMP in DC monotherapy, TYMP expression in CT-26 tumor tissues (**Figure S2D**) was compared and correlated (multivariate correlation analysis) with the number of CD31+ and CD8+-PD1+ cells among TILs and the mean survival among different groups. TYMP was thoroughly expressed in tumors and was positively correlated with CD31 expression (r=0.6502) (**Figure S2F**). Similarly, significant associations were observed between TYMP expression and CD8/PD1 (r=0.70803) (**Figure S2F**). This further revealed that high TYMP expression was associated with a significantly lower survival rate than low TYMP expression.

### Targeting TYMP does not prolong CRC survival but induces ICD, preventing T-cell exhaustion and mature immune effector cells

**Figure 3:**
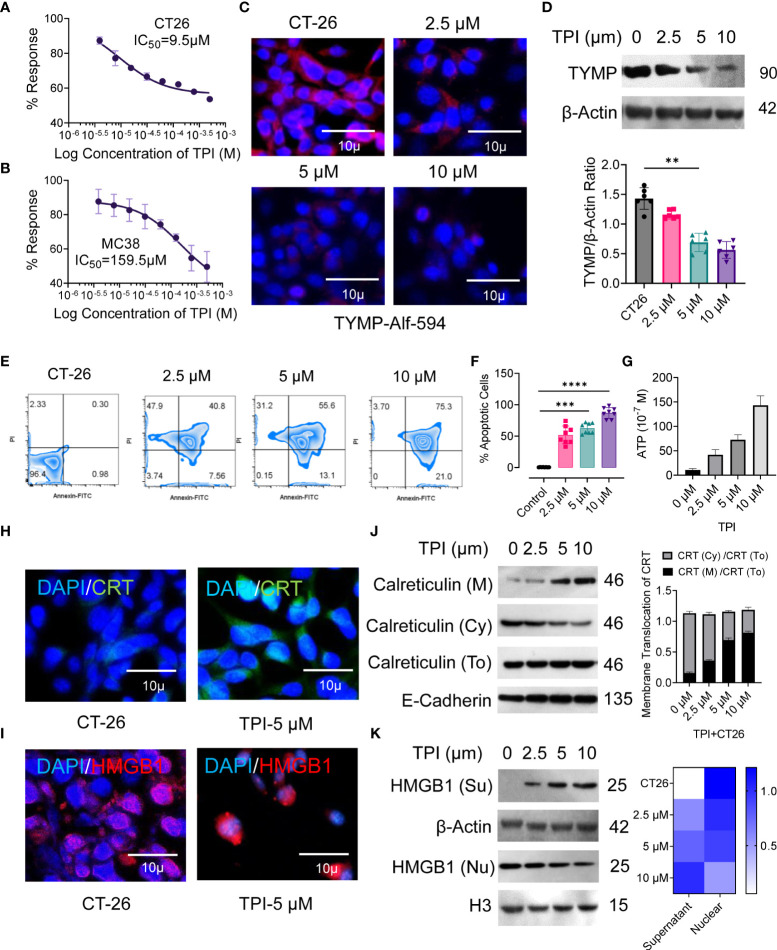
Inhibition of thymidine phosphorylase (TYMP) *in vitro* with tipiracil hydrochloride (TPI) potentiating cytotoxicity and ICD induction in colorectal cancer cells. The *in vitro* antiproliferative effects of TPI were studied using a 48-h exposure of the murine colon cancer cell lines **(A)** CT-26 and **(B)** MC-38. **(C)** CT-26 tumor cells were treated with increasing concentrations of TPI for 8 h at 37°C and 5% CO_2_. The cells were washed in PBS (×2), stained with PE-labeled anti-TYMP antibody for 2 h and observed under a microscope (Leica DMi8). A representative image of 3 similar experiments is shown. **(D)** TYMP expression in untreated and TPI-treated CT-26 cells was examined by Western blot. β-actin was reblotted as a loading control. **(E, F)** Annexin V staining for the assessment of percent apoptosis in CT-26 tumor cells in the presence of varying concentrations of TPI. Representative zebra plot of eight independent experiments. **(G)** ATP release by dying tumor cells is the hallmark of ICD. ATP release into the supernatant of CT-26 cells treated with TPI (mean ± SD, n=4). **(H, I)** Translocation of CRT from the cytosol/ER (Cy) to the cell surface (M) and relocalization of HMGB1 from the nucleus of CT-26 cells after incubation with or without TPI (5.0 µM). **(J, K)** Western blot analysis of membrane CRT and nuclear (nu) and extracellular (Su) localization of HMGB1 in CT-26 cells after treatment with varying concentrations of TPI. Western blot gray value of HMGB1 and CRT protein intensity (n = 6 independent replicates). For the violin plot, the middle line is the median, the lower and upper hinges correspond to the first and third quartiles, and lover and upper adjacent values represent ±1.5 interquartile ranges. Mean densiometric quantification of bands as indicated (column-scaled z scores) fractions for HMGB1. The color scale indicates the relative expression levels. Unless otherwise noted, the data are presented as independent biological triplicates. *In vivo* studies were repeated independently five times to confirm the results. **p < 0.01, ***p < 0.001, and ****p < 0.0001.

Currently, fluoropyrimidine-based drugs, such as 5Fu, are the first choice of drugs for the treatment of gastrointestinal (GI) tract cancers, but building resistance to 5Fu remains a major issue (4). For third-line treatment of chemorefractory metastatic colorectal cancer, the oral chemotherapeutic agent TAS-102 (also known as trifluridine-tipiracil, which includes TPI) was recently approved in the USA, JAPAN, and Europe, and several investigations are ongoing in different combination settings (26, 36). Tipiracil is an orally administered active TYMP inhibitor. We found that TYMP overexpression in CT-26 tumors was associated with an immunosuppressive TME and poorer survival, even with DC-based vaccination (Figs. 1 and 2). Therefore, we tested the effect of TYMP silencing on the CT-26 TME. The in vitro tumoricidal activities of TPI against murine CRC tumor cells CT-26 and MC38 showed concentration-dependent growth inhibition with IC50 values of 9.5 µM (CT-26) and 159.5 µM (MC38) (**Figures 3A, B**). We selected doses of 2.5, 5, and 10 µM, with the latter inducing approximately 50% CT-26 cell death (**Figure 3A**). TPI inhibited TYMP expression in CT-26 cells (**Figures 3C, D**) in a concentration-dependent manner. Moreover, TPI also induced apoptosis in CT-26 cells (**Figures 3E, F**), indicating that TPI concurrently downregulated the expression of TYMP and inhibited the growth of CT-26 cancer cells.

Several studies have demonstrated that ICD is a molecular process induced by anticancer agents that triggers a CTL-dependent adaptive immune response (37). Cancer cells undergoing ICD require cell surface calreticulin (CRT) exposure, induction of EIF2α-dependent reticulum stress, high mobility group box 1 (HMGB1) and ATP release, and the expression of type 1 IFNs (Ifnα1 and Ifnβ1) and chemokines (Cxcl 9 and Cxcl 10) (38, 39). Therefore, we validated the ability of TPI to induce hallmarks of ICD in CT-26 cells in vitro, as confirmed by the increased CRT cell surface translocation (**Figures 3H, J**) and release of damage-associated molecular patterns (DAMPs), such as ATP (**Figure 3G**) and HMGB1 (**Figures 3I, K**), compared to the untreated control. Upon TPI treatment, HMGB1 translocated from the nucleus to the cytoplasm and was released into the extracellular compartment (**Figures 3I, K**). Overall, these data showed that TPI alone can induce ICD in vitro in CT-26 cell lines that are resistant to immunotherapy. ICD converts tumor cells into “tumor vaccines” by promoting tumor cell recognition and processing by DCs in vivo (40). To demonstrate that TPI-treated CT26 tumor cell-derived DAMPs could be effectively captured by DCs, we vaccinated BALB/c mice with either TPI-treated or heat-killed CT26 cells, followed by subcutaneous rechallenge of CT26 cells in vaccinated mice. Tumor growth was monitored. As shown in **Figure S4**, mice vaccinated with TPI-treated CT26 cells displayed greater tumor growth retardation (**Figures S4B–E**). Ex vivo analysis of DC activation was further established by the increased expression of the costimulatory molecules CD80 and CD86 and an antigen-specific DC-mediated immune response (**Figures S4F, G**).

**Figure 4:**
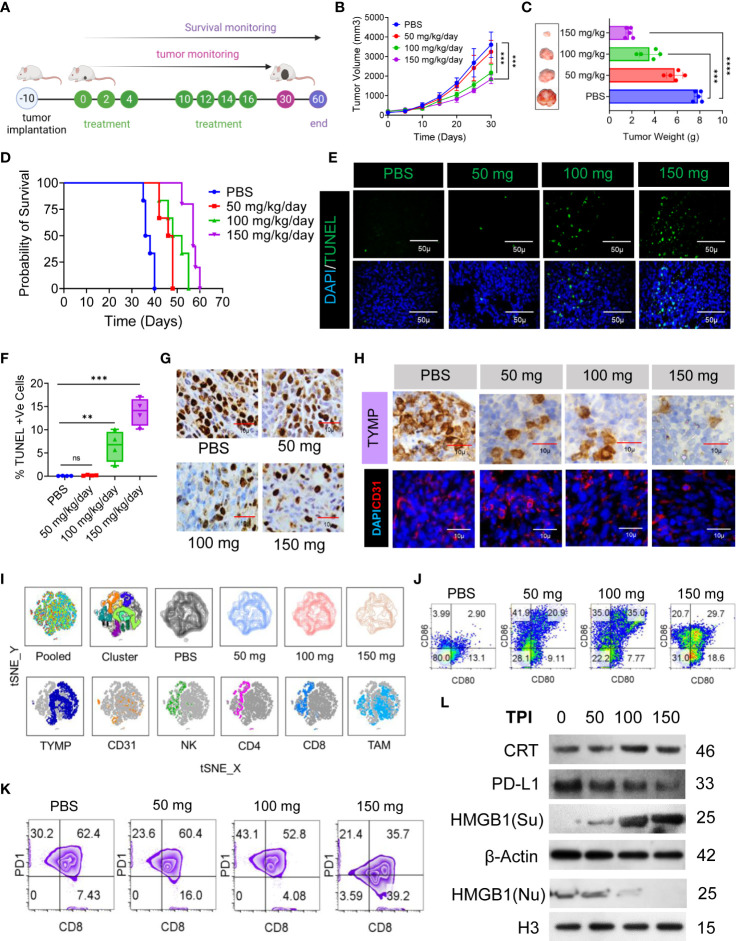
TPI monotherapy does not prolong CRC survival but induces ICD or downregulates PD-L1 in CRC cells, and ICD-associated DAMPs mediate DC maturation. **(A)** Schematic representation of the experimental setup to evaluate the effect of TPI monotherapy on the survival of CT-26 CRC-bearing mice (n = 6) (four independent repeats of survival assessment). **(B)** Tumor growth curves (n=6). **(C)** The tumor weight (mean ± SD, n=6) of each group and representative images of the tumors dissected on day 30; n = 3 mice. **(D)** Kaplan–Meier analysis of the mice; n = 6 mice. Median survival for PBS (37 days), 50 mg kg^-1^ bw (47 days), 100 mg kg^-1^ bw (50 days), and 150 mg kg^-1^ bw (57 days), p = <0.0001]. **(E)** TUNEL staining of the tumor sections dissected on day 30; green: TUNEL; blue: DAPI; scale bars: 50 μm; to confirm the results, the experiment was repeated independently three times. **(F)** Boxes represent interquartile ranges (IQRs) of the percentage of TUNEL-positive cells. The experiment was performed in triplicate with four mice, and the results are presented as the mean ± SD. **(G)** Representative tumor tissue sections from three mice showing immunostaining for Ki-67. The bar indicates 10 µm. **(H)** IHC analysis of TYMP (top) and immunofluorescence analysis of CD31 (bottom) in CRC tumors at day 30 of the indicated groups. **(I)** Global tSNE projection of TILs in the polled and indicated treatment groups (upper panel) and tSNE maps showing the expression level and the distribution of each indicated marker among the identified clusters (lower panel). TYMP (deep blue), CD31 (orange), NK cells (green), CD4^+^ T cells (pink), CD8^+^ T cells (light blue) and TAMs (sky blue) in TILs for all treatment groups pooled, concatenated and overlaid. **(J)** ICD-associated DAMP-mediated DC maturation determined by flow cytometry (CD11c^+-^gated CD80^+^CD86^+^), n=3. **(K)** Representative PD1 expression in CD3^+^/CD8^+^-gated TILs. **(L)** Western blot analysis of CRT, HMGB1 and PD-L1 expression in tumor tissue and its fold increase or decrease compared to the untreated control. β-Actin and H3 were used as loading controls for the cytoplasmic and nuclear fractions, respectively, n=3. Unless otherwise noted, the data are presented as independent biological triplicates. *In vivo* studies were repeated independently five times to confirm the results. ns, not significant, **p < 0.01, ***p < 0.001, and ****p < 0.0001.

Having established that ICD is specifically induced by TPI in the CT-26 cell line, we evaluated the antitumor potential of TPI against established CT-26 tumors. TPI was administered orally (diluted in HPMC) according to the schedule presented in **Figure 4A** at the reported optimal effective dose (150 mg/kg/day) (41) and two lower doses (100 and 50 mg/kg/day). The control groups received 10 mL/kg vehicle (0.5% HPMC solution) orally (**Figure 4A**). Dose-dependent antitumor activity, characterized by decreased tumor burden (**Figures 4B, C**) and increased survival (**Figure 4D**), was observed starting at 50 mg/kg/day TPI compared to the untreated CT-26 control (p<0.05). Additionally, the reported optimal effective dose of TPI led to significant tumor regression and increased survival for up to 60 days (**Figure 4D**). To explore whether any phenotypic changes occurred due to TPI treatment in CT-26 tumor tissues, the percentages of apoptotic and proliferating cells were examined. The TUNEL assay revealed that in the 150 mg/kg/day group, more (13.87 ± 2.95, n=4) TUNEL-positive cells were identified, which was a 3-fold increase compared to the levels seen in the 50 mg/kg/day-treated group (0.185 ± 0.091, n=4, P < 0.05; **Figures 4E, F**). Ki-67 staining showed fewer proliferating cells (brown) in tumor tissues treated with a high dose (126 ± 15.3, n=8, Ki-67+ cells/field) than in those treated with 50 mg/kg/day (447 ± 28.68, P < 0.05; **Figure 4G** and **S5A**) or the control group (642 ± 15.59, P < 0.01). Furthermore, analysis of TYMP and CD31 expression in tumor tissues indicated a significant reduction in TYMP-dependent neoangiogenesis (**Figures 4H**, **S5B, C**). Immunophenotyping of the TILs by multiparametric flow cytometry followed by pooled and individual t-SNE analysis revealed 12 different clusters of unique subpopulations with a significant increase in infiltrating CD4+ and CD8+ T cells after low-dose TPI (50 mg/kg/day) treatment (**Figure 4I** and **S5D, E**). Significantly increased NK-cell (CD49+) and DC infiltration (CD11c+/Class II) and expression of the costimulatory molecules CD80 and CD86 indicated increased DC activation (**Figures 4I, J** and **S5F, G**). Surprisingly, in the case of low-dose TPI treatment (50 mg/kg/day), we observed a large amount of DC infiltration and, in the case of effective TPI treatment (150 mg/kg/day), a significant reduction in CD8+PD-1+ cell numbers (**Figure 4K**) compared with the untreated control. Moreover, consistent with ICD induction, TPI treatment led to a reproducible increase in CRT and HMGB1 expression and downregulated PD-L1 expression in tumors in vivo (**Figures 4L** and **S5 H**). The released DAMP molecules are present in the immune system for recognition and processing by DCs. Thus, ICD-induced DC infiltration and maturation were observed in vivo using splenic DCs isolated from BALB/c mice in different treatment groups (**Figure 4J**). To examine potential TPI toxicity, vital organs, including the liver, lung, spleen, and kidney, were dissected and H&E-stained for histopathological analysis, with no significant pathological changes observed (**Figure S5I**). The liver and lungs are the two most frequent sites of metastatic CRC. Histological assessment of the metastatic burden in the liver and lungs revealed multiple metastatic regions in the controls. In contrast, the liver and lungs from animals receiving TPI treatment showed few peripheral nodules of small sizes, with a reduced metastatic region (**Figure S5I**). We also assessed the toxic effect of TPI on DC/IMQDC and found that suboptimal (half of the IC50 used for analysis) doses of TPI had no significant effect on IMQDC viability (**Figure S5J**). Moreover, a lower percentage of necrotic area was observed in tumor tissues following H&E staining in all the TPI-treated groups (**Figures S5K–L**). Combined, this evidence and the multivariate correlation analysis of the studied factors (**Figure S5 M**) suggest that targeting TYMP with TPI does not significantly prolong the survival of CT-26 tumor-bearing animals but induces ICD at all in vivo doses of TPI tested. TPI treatment generates antitumor effects by activating immune cells via ICD-induced DAMP signals, thereby inducing tumor cytotoxicity and cellular apoptosis.

### Chemoimmunotherapy induced a higher level of ICD than either treatment alone and abolished immunotherapy resistance induced by TYMP overexpression

Although TPI monotherapy induces ICD in vivo and activates a certain degree of DC activation, neither IMQ-DC nor TPI monotherapy achieved a significant antitumor response in solid CT-26 tumors in terms of survival. Therefore, to achieve a deeper response against CRC, we combined two nonoverlapping treatments, that is, a low nontoxic chemotherapy regimen and DC-based adoptive cell therapy.

**Figure 5:**
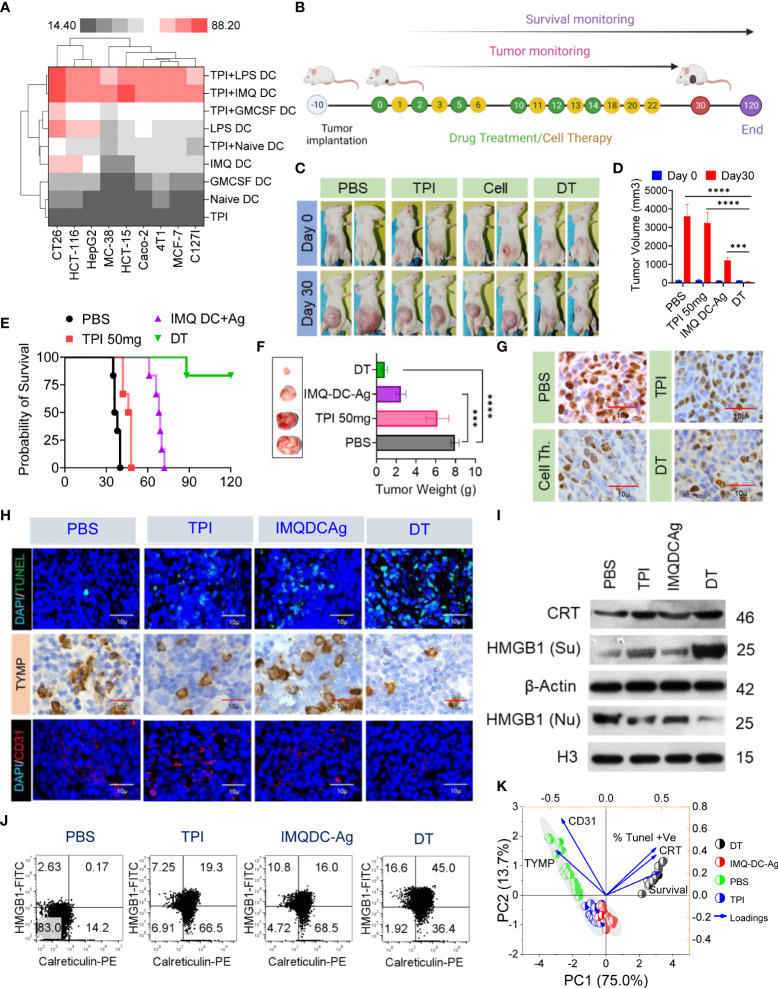
Extended tumor-free survival and enhanced antitumor immunity against CRC in mice treated with tipiracil + imiquimod-activated DCs are accompanied by higher levels of ICD. **(A)** Heatmap showing the enhancement of growth inhibition of TPI-treated murine (CT-26, MC38, 4T1, C127i) and human (HCT116, HCT-15, HepG2, Caco2, MCF-7) cells by GMCSF (1000 U/ml) and imiquimod (200 pg/ml)-activated murine BMDCs and human DCs. LPS (10 μg/ml) was used as a positive control. **(B)** Experimental schedule; treatments as in **(A)** every alternate day 6 times with two booster doses of cell therapy. **(C)** Images of the animals in the indicated groups. **(D)** The averaged tumor growth diagram at day 0 *vs* day 30 in the indicated groups. **(E)** Kaplan–Meier analysis of the mice; n = 6 mice. Median survival for PBS (37 days), 50 mg kg^-^1 bw/day (47 days), IMQDC+Ag (70 days), and dual therapy (DT) (undefined), p <0.0001]. **(F)** The average tumor weight of each group and images of the tumors dissected on day 30; mean ± SD, n = 3 mice. **(G)** Representative tumor tissue sections from three mice showing immunostaining for Ki-67. **(H)** TUNEL staining of the tumor sections dissected on day 30 (top); green: TUNEL; blue: DAPI; IHC analysis of TYMP (middle), and immunofluorescence analysis of CD31 (bottom) in CRC tumors of the indicated groups, scale bars: 50 μm; the experiment was repeated independently three times to confirm the results. **(I)** Western blot analysis of CRT and HMGB1 expression in tumor tissue and its fold increase or decrease compared to the untreated control. β-Actin and H3, loading controls for the cytoplasmic and nuclear fractions, respectively. **(J)** Dot plot represents flow cytometric analysis of CRT-HMGB1-positive cells (gated on propidium iodide-negative cells (PI)). **(K)** Principal component analysis to predict the biomarker correlated with median survival among treatment groups. Unless otherwise noted, the data are presented as independent biological triplicates. *In vivo* studies were repeated independently five times to confirm the results. and ***p < 0.001.

First, we tested the in vitro cytotoxic effect of suboptimal (half of IC50) doses of TPI or activated BMDCs/human peripheral blood DCs alone or in combination on a variety of murine (CT-26, MC38, 4T1, and C127I) and human (HCT116, HCT15, Caco2, HepG2, and MCF7) cell lines. Following pretreatment with a lower concentration of TPI (5 µM), the addition of GM-CSF DCs or activated (IMQDCs or LPSDCs) murine BMDCs and human peripheral blood DCs demonstrated synergistic tumoricidal effects against all the cell lines tested (**Figure 5A**), and TPI potentiated DC-mediated tumoricidal effects. IMQ-activated DCs showed enhanced tumoricidal activity against CT-26 and HCT15 cells when used in combination with TPI (43.21 ± 1.6 vs. 53.95 ± 6.92, n=12, between IMQ DCs alone and combined treatment with IMQDCs+TPI). The column-scaled z score analysis clearly demonstrated that the combination treatment had an advantage over the IMQDC-only treatment (**Figure 5A**). The in vivo antitumor activity of the combination of TPI (50 mg/kg/day) and IMQDC-Ag was evaluated, as shown in **Figure 5B**. TPI clearly manifested no significant tumor inhibition ability, and IMQDC-Ag arrested tumor growth initially and later regained growth momentum (**Figures 5C, D**). However, combined treatments (DT) immediately caused the tumors to gradually regress, even after the last therapy, until all the tumors were extirpated (complete response, CR) (**Figures 5E, F**). In the follow-up observation, no relapse was observed, and all treated animals were very healthy, even at the end of the experiment, 120 days after the first therapy. Eight days after the final treatment, three animals from each group were sacrificed for immunofluorescence and histopathological analyses. The tumor sections were analyzed using Ki-67 (**Figure 5G**, TUNEL, TYMP, and CD31 staining (**Figure 5H**). Dual-treated tumors contained few proliferative (Ki-67+) cells (**Figures 5G** and **S6D**), increased apoptotic cells, and significant downregulation of TYMP and CD31+ cells compared with either treatment alone or the untreated control (**Figure 5H**). Histological assessment of vital organs revealed no significant pathological changes. In the DT group, no metastatic burden was observed, whereas the liver or lungs from animals receiving either treatment alone showed a few small metastatic regions (**Figure S6A**). The absence of a central necrotic area in the DT group compared with that in the untreated group and treatment with TPI or IMQDC-Ag alone (**Figures S6B, C**) immediately caused the tumors to gradually regress. We assessed the in vivo capacity of the combined treatment to induce ICD. The highest CRT signal intensity (2.8-fold) of the tumors treated with DC alone (**Figures 5I, J**, **Figure S6E**) was observed in the tumors treated with DT. Notably, TPI enhanced CRT expression to a higher level than that in the untreated and IMQDC-Ag alone groups but had no antitumor potential (**Figure S6F**). Upon DT treatment, HMGB1 translocated from the nucleus to the cytoplasm, and only the combination of TPI and IMQDC-Ag significantly induced HMGB1 cytoplasmic relocalization (**Figures 5I**, **S6 G, H**). Despite the absence of HMGB1 cytoplasmic relocalization and CRT expression, IMQDC-Ag monotherapy had an antitumor effect in immunocompetent CT-26 mice. These observations suggest an ICD-independent immune effect of IMQDC-Ags, such as the exhaustion of an immunosuppressive population (**Figures 2I, K**). Finally, we performed principal component analysis (PCA) to examine the potential mechanisms associated with CR in chemoimmunotherapy. DT-enhanced survival, CRT expression, and apoptosis of tumor cells (TUNEL+) were clearly separated from TYMP expression and CD31+ cells in the PCA space and were associated with tumor growth, whereas TPI and IMQDCs largely overlapped (**Figure 5K**). PCA further confirmed that combined therapy abolished the immunotherapy resistance induced by TYMP by triggering ICD in vivo.

### Deep immune profiling reveals that chemoimmunotherapy reshapes the TME, prevents T-cell exhaustion and converts Treg cells to CD4+ T effector cells

**Figure 6:**
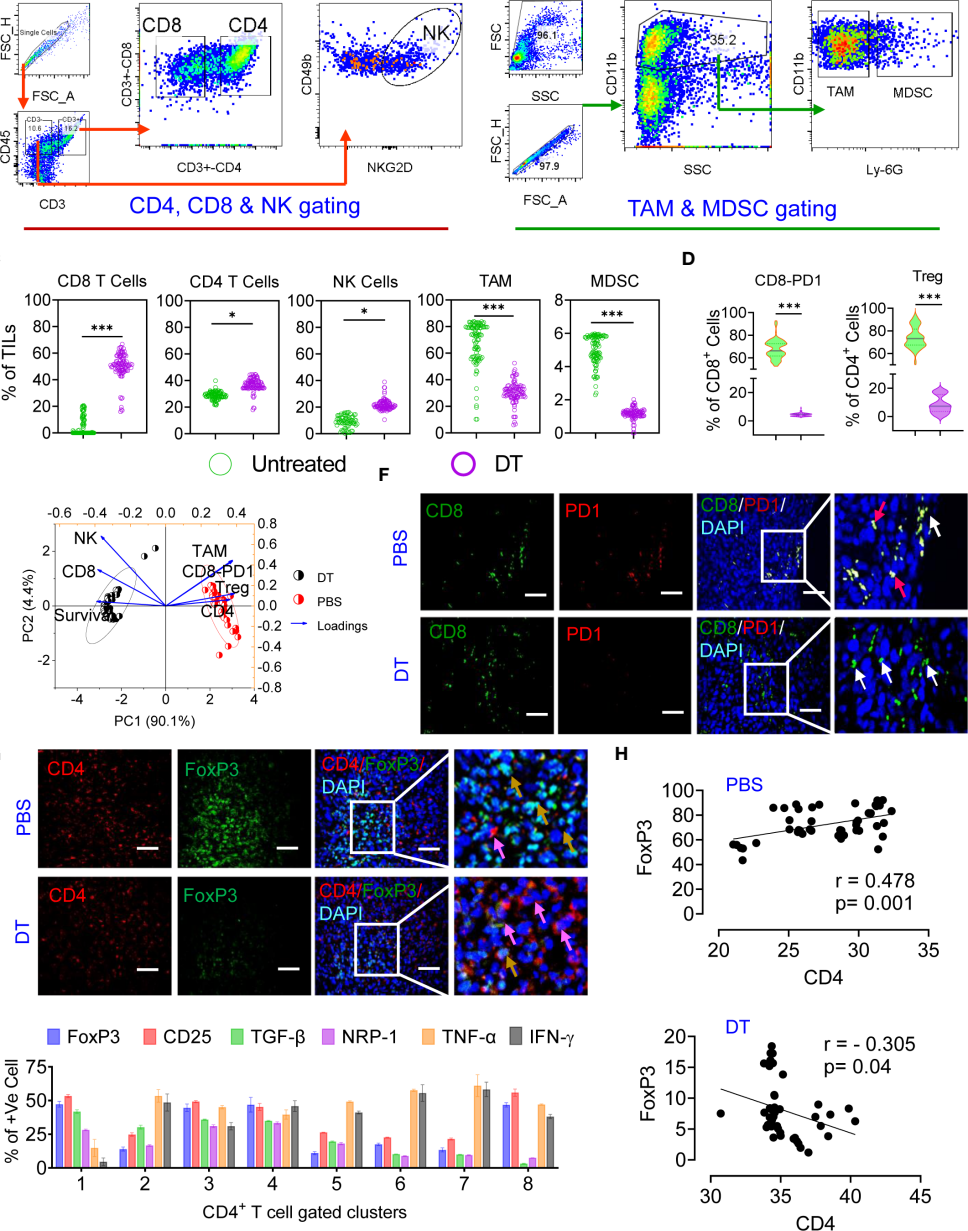
Chemoimmunotherapy prevents CD8 T-cell exhaustion and converts Treg cells to CD4 T effector cells. Gating strategy of CD4, CD8, NK cells **(A)** and TAMs and MDSCs **(B)** in TILs. The percentages of CD8^+^ T cells, CD4^+^ T cells, NK cells, TAMs and MDSCs in the percentage of TILs among the identified clusters are presented in **Figure S7** **(C)**. Percentage of PD1^+^ and Foxp3^+^ cells among the CD8^+^ and CD4^+^ populations in the treated *vs*. DT groups **(D)**. Principal component analysis to predict the different immune cell populations in TILs correlated with median survival among treatment groups **(E)**. Immunofluorescence localization of CD8^+^ (green, white arrow) or CD8^+^/PD1^+^ T cells (yellow, red arrow) **(F)** and CD4^+^ (red, pink arrow) or CD4^+^/Foxp3^+^ dual-positive Treg cells (yellow arrow) in the tumor tissue of the untreated and dual-treated groups, scale bar 10 µM **(G)**. A representative image of 3 similar experiments is shown. Linear regression and correlation analysis of CD4 *vs*. Foxp3 cells among the untreated (r=0.478, p=0.001) and dual-treated (r= -0.305, p=0.04) groups **(H)**. The percentages of Foxp3, CD25, TGF-β, NRP-1, TNF-α and IFN-γ among the identified clusters are presented in **Figure S7** g **(I)**. All p values were calculated using one-way ANOVA and corrected for multiple comparisons using Tukey’s adjustment. Unless otherwise noted, the data are presented as independent biological triplicates. *p < 0.05, and ***p < 0.001.

An effective antitumor immune response induced by chemoimmunotherapy is advanced by the capacity of chemotherapies to alter the immunosuppressive TME and/or instigate ICD, resulting in successive T-cell recruitment (37). Thus, we studied the effect of each therapy regimen on immunosuppression and the effector immune response. To gain a better understanding, we carried out deep immunoprofiling and IF microscopy of TILs, followed by global high-dimensional mapping of multiparameter flow cytometry data. A tSNE representation of the data emphasized the key regions of CD4 (pink) CD8 (orange) T cells and NK cells (blue) found preferentially among treatment groups (**Figure S7A**). Compared with untreated mice, only IMQDC-Ag and chemoimmunotherapy (DT) induced an increase in CD8+ and CD4+ T cells among immune cells, whereas the NK-cell fraction increased with TPI treatment alone (**Figures 6A–D**, **S7A** lower panel). Principal component analysis to predict the different immune cell populations in TILs correlated with median survival among treatment groups and showed that an increased number of CD8+ and NK cells was positively correlated with a better prognosis upon DT (**Figure 6E**). Importantly, immunofluorescence analysis of tumor samples showed that the combination therapy promoted CD8+ T-cell infiltration not only in the tumor periphery but also in the tumor core (**Figure 6F**) and diminished Treg numbers (**Figures 6D, G, H**). There was a notable increase in the density of tSNE regions that mapped to the expression of IFN-γ, GrB, ROS, and TNF-α (**Figure S7B**). FlowSOM clustering was performed, and 10 CD8+ T-cell markers were compared in each identified cluster (**Figures S7C, D**). This approach identified that DT could enhance GrB, IFN-γ, and TNF-α production in intratumoral CD8+ T cells, whereas monotherapies induced the production of only one or two cytokines in several clusters, including clusters 1, 4, 5, and 7. Clusters 6 and 8 contained CD69+, PD-1+ and TIM3+ activated, proliferating cells and were more prevalent in the untreated or monotherapy groups (**Figures S7B–D**).

A significant number of research articles have mainly focused on the CD8+ T-cell response during immunotherapy, while the antitumor efficacy of CD4+ T cells has not been fully studied (42). Combined treatment with TPI and IMQDC-Ag also significantly increased CD4+ T-cell infiltration (**Figure 6C** and **S7A**). Global CD4+ T-cell differentiation patterns were projected into the tSNE space in CRC tumors compared to IMQDC-Ag monotherapy and chemotherapy (**Figures S7E, F**). In untreated CT-26 tumor-bearing animals, there was a notable increase in the density of tSNE regions that mapped to the expression of FoxP3, CD25, TGF-β, and NRP-1 (**Figure S7F**). We used a FlowSOM clustering approach to gain more insight into these changes in CD4+ T-cell cells (**Figures S7 G, H**). FlowSOM clustering identified an increase in clusters 1, 8, and 3 (indicating the populations that express CD25, FoxP3, TGF-β, and NRP-1) in untreated CT-26 tumors compared to TPI and IMQDC-Ag alone or in combination (**Figures S7 G, H** and **Figures 6G–I**). In contrast, this clustering approach identified a significant decrease in regulatory T cells (clusters 2 and 3; **Figure S7 G**) in the tumor compared with the untreated control.

### Binary therapy converts CRC intratumor Treg cells to Th1 effector cells, leading to reduced tumor growth in mice

**Figure 7:**
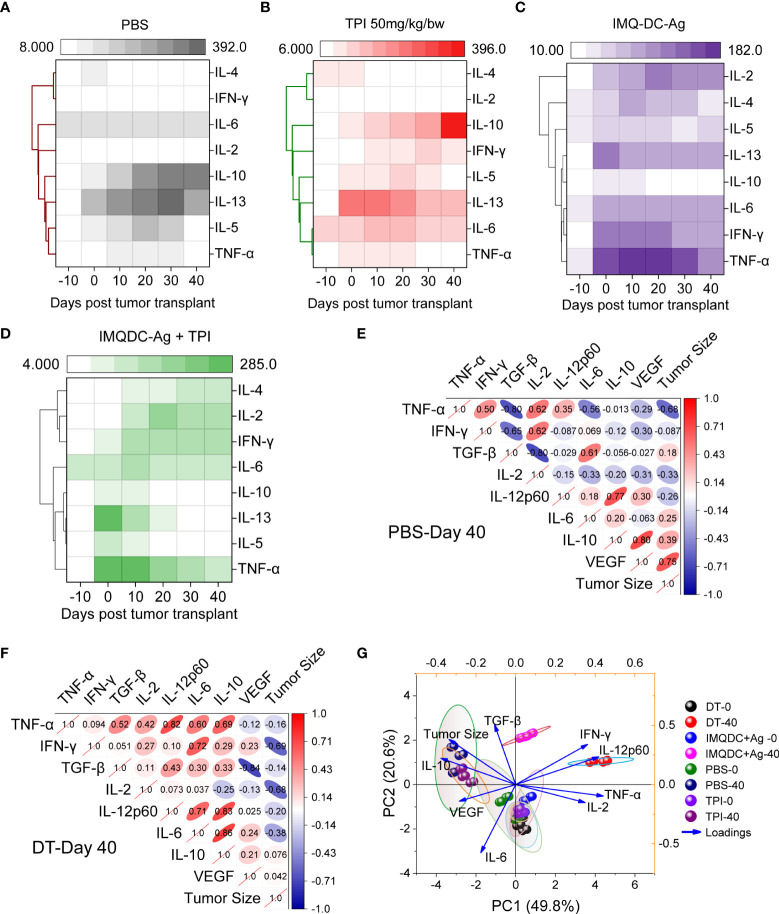
Development of Th1-type humoral responses leading to enhanced survival in mice. An 8-plex bead-based multiplex analysis of the Th1/Th2 panel was performed for **(A)** CT-26 untreated, **(B)** TPI, **(C)** IMQDC-Ag and **(D)** TPI+IMQDC-Ag. A heatmap with a dendrogram on the side shows the clustering of cytokines. The colors in the heatmap represent cytokine expression intensities scaled to the mean of zero and unit variance for each cytokine. Cytokine expression in serum samples detected by ELISA and correlation matrix graph of Pearson’s correlation coefficients of the indicated cytokine secretion levels in the untreated group **(E)** *vs*. dual-treated group **(F)** at day 40. PCA of various cellular and humoral immune profiles regulated during the vaccination periods was performed to predict biomarkers for long-term immune protection **(G)**. Unless otherwise noted, the data are presented as independent biological triplicates.

The major contributing CD4+ T cells in tumor immunology are subdivided into different subsets, that is, Th1, Th2, Th17, and Tregs, based on their secretory cytokines and immunological roles (43). IL1β, IL-2, IL-12, TNF-α, and IFN-γ, known as Th1 cytokines, are associated with good prognosis in many malignancies, whereas Th2 cytokines (IL-4, IL-5, and IL-10) are associated with tumor growth and metastasis (44, 45). Tregs are generally immunosuppressive and are correlated with CRC progression (**Figures S7E–H**), and there is uncertainty regarding the role of Th17 cells. To examine the potential role of Th1 and Th2 cytokines and Treg responses in predicting the prognosis of CT-26 tumors treated with chemoimmunotherapy. We performed a bead-based multiplex analysis of the Th1/Th2 panel and ELISA analysis of plasma and serum samples from different treatment groups (**Figure 7**). Higher levels of IL-10, IL-4 (**Figure 7A** and TGF-β (**Figure 1I**) production in untreated CT-26 tumor-bearing animals strongly correlated with Treg induction and CRC progression, as we observed in **Figure 1**. Treatment with combination therapy increased the plasma concentrations of IFN-γ, TNF-α, IL-2, and IL-6, indicating that tumor-infiltrating Treg cells gained CD4 effector function (**Figure 7D**). Similar to the untreated control (**Figure 7A**), TPI monotherapy enhanced Treg induction (**Figure 7B**). Treg cells in the IMQDC-Ag-treated group showed a CD4 effector phenotype similar to chemoimmunotherapy, as opposed to the induced Treg cells observed in the tumor control and following TPI treatment (**Figure 7C**). Associations between the levels of T-cell cytokines or Tregs and tumor size, which represents tumor prognosis after chemoimmunotherapy, were investigated. Multivariate correlation of ELISA data (different cytokines) among untreated (**Figure 7E**) vs. dual-treated (**Figure 7F**) groups on day 40 (when the first individual of the untreated group died) revealed that early tumor growth was positively correlated with IL-10 (r=0.39), VEGF (r=0.75), and TGF-β (0.18), whereas the CR of tumors in the DT group was largely correlated with Th1 cytokines. Next, we performed PCA of various cellular and humoral immune profiles regulated during the vaccination period to identify biomarkers for long-term immune protection. Tumor growth retardation was strongly correlated with IFN-γ and IL-12 responses (**Figure 7G**).

### IFN-γ-mediated cytolysis and MHC-dependent recognition are required for the antitumor activity of reactivated CD8 T cells following chemoimmunotherapy

IFN-γ directly affects tumor cells by decreasing proliferation, downregulating metabolic activity, enhancing costimulatory molecule and MHC expression, and inhibiting angiogenesis through the induction of CXC chemokines (46). Although CT-26 tumors are known to be highly immunogenic owing to the high expression of major histocompatibility complex I (MHC I) molecules, an absence of tumor-infiltrating CTLs and mature DCs was observed in CT26 tumors (47). Therefore, we were interested in determining the mechanism of MHC induction in CT-26 tumors, which renders them susceptible to T-cell cytotoxicity. In our previous study, we showed that DT predominantly produced the effector cytokine IFN-γ by T cells (**Figures 6**,**7**), which upregulated surface MHC expression (48). Thus, we hypothesized that T-cell-derived IFN-γ might induce CT-26 tumors to increase surface MHC expression. We found that CT-26 tumor cells upregulated both MHC I and II upon stimulation with IFN-γ in vitro in a dose-dependent manner (**Figures S8A, B**). Consistent with our in vitro observations, DT selectively upregulated MHC class I and II. Collectively, chemoimmunotherapy upregulated MHC I and II molecules in tumor cells (**Figures S8C, D**). To test whether CD8 T cells recognize and kill target cells via MHC I and II, we tested the cytotoxic potential of CD8 T cells against CT-26 tumors in the presence or absence of blocking antibodies for MHC I and II. As expected, MHC I blockade abrogated the antitumor effect of CD8+ T cells (**Figure S8E**). This suggests that a direct TCR-MHC interaction is needed for the antitumor effects of T cells. Since we observed maximum tumor growth retardation in mice through the cooperation of CD8+ T cells, we investigated whether direct cell contact is required for tumor cell killing. Thus, we compared the viability of CT-26 cells in direct contact with CD8+ T cells or CT-26 cells separated from CD8+ T cells using a Transwell membrane (**Figure S8F**). We observed reduced tumor cell viability in the Transwell setup, suggesting that the optimal T-cell-mediated killing of CRC tumors is contact dependent. We also established that IFN-γ is required for killing CT-26 cells, as we observed no cytotoxicity against tumor cells upon IFN-γ blockade in Transwell assays (**Figure S8F**). We also investigated whether the cytotoxic activity of CD8+ T cells upon combined treatment was dependent on granzyme B, TNF-α, and IFN-γ. Neither TNF-α nor GzmB blockade abrogated the therapeutic efficacy of the combined treatment (**Figure S8F**)

## Discussion

A multifactorial mechanism of cancer resistance to immunotherapy has been reported in the literature, and the immunosuppressive TME plays a critical role in the resistance of CRC to immunotherapies or ACTs. Overexpression of TYMP in CRC or other malignancies and TYMP induced increased secretion of IL-10 and TGF-β in the TME, which inhibited the effector functions of DCs and increased CD8 T-cell exhaustion and conversion of effector T cells to Tregs, which are thought to be responsible for the creation of an immunosuppressive TME (19). TYMP is responsible for systemic tumor-specific CD8+ cell exhaustion and abrogates adoptive dendritic cell therapy efficacy in a preclinical CRC model, which depicts systemic T-cell loss and reduced ACT or immunotherapy efficacy observed in animal models with advanced CRC. Therefore, TYMP has been the target for alleviating immunosuppression in CRC tumors (20, 21, 23–25). Here, we found that tipiracil hydrochloride, a small molecular inhibitor of TYMP, induces an ER stress response that ultimately leads to the production of DAMPs (CRT, HMGB1, ATP) and primes ICD in vitro and in vivo in an experimental model of CRC. Upon targeting TYMP with a reported optimal effective dose of TPI (150 mg/kg/day), in addition to suppression of TYMP expression in tumor tissue, ICD could be induced in vivo but simultaneously caused damage to the host immune system (**Figures 4J, K**) and was unable to generate a better prognosis; withdrawal of therapy regained tumor growth momentum. While a low or suboptimal dose of TPI is used to prevent chemotherapy-induced immune suppression and nontarget damage to the hosts, we observed substantially lower CRT exposure, higher PD-L1 expression in tumors but more effector TILs (CTLs and NKs), and fewer exhausted CD8 T cells, Tregs, and TAMs. Our observation is in line with earlier reports (40, 49), in which DAMPs released by dying tumor cells due to ICD can promote functional DC maturation and activation, and greater T-cell-mediated antitumor activity in immune competence is observed. Despite the efficient ICD inducer, a suboptimal dose was unable to generate a better prognosis due to higher PD-L1 expression in tumors, which may intern convert effector T cells to suppressor T cells in the long run.

In addition to chemotherapy, there is a significant interest in the design of alternative therapeutic strategies that can break tumor tolerance and generate strong antitumor immunity. In addition to ICBs, considerable interest has been placed on strategies that engage critical components of the host immune system that bridge innate and adaptive immunity. One such system is the TLR system. Although a proof of principle for this concept has been proposed with agonists of several TLRs (3, 7, and 9), only one TLR7 agonist (imiquimod) has been approved for clinical use. However, in addition to the promise of such TLR agonist-based therapies, the pharmacological use of TLR agonists itself has not been borne out in the clinic due to “cytokine storm”-related dose-limiting toxicities (34). With the idea of combining one plus one to obtain four, we investigated the potential of an original combination of the thymidine phosphorylase (TYMP) inhibitor tipiracil hydrochloride (TPI) and TLR7 agonist (imiquimod)-activated DC therapy in a CT26 syngeneic immunocompetent colorectal cancer mouse model CT-26 tumors (microsatellite stable, MSI-L, and Kras-mutant) (33), compared them with those treated with monotherapies and healthy individuals, and performed integrated analysis of immune features. The rationale for combined therapy is that TPI downregulates TYMP and turns tumor cells immunogenic via induction of immunogenic cell death (ICD) to present tumor antigens to DCs to recruit T cells and modulate tumor-infiltrating lymphocytes (TILs). Ex vivo stimulation of DCs with a TLR7 agonist (imiquimod) leads to increased cytokine secretion and elevated expression of costimulatory molecules (CD40, CD70, and CD86) (50). These cytokines prime NK cells and T cells, leading to their improved activation. While exogenous TLR7 activates DCs, it disrupts immunotolerance and restores T-cell homeostasis in an additive manner. Therefore, combining two nonoverlapping anticancer mechanisms may achieve a deeper response against pMMR-MSI-L-type colorectal cancer.

Murine CT-26 cells, similar to human CRC cells, are massively invaded by TAMs and Tregs, which are associated with poor prognosis (51). In our study, we observed that neither a low dose of TPI nor IMQDC-Ag alone could induce ICD in vivo, whereas combination therapy did. This difference may be explained by the synergistic effect of chemotherapy with DC therapy, although it is difficult to determine whether it is additivexor truly synergistic. Multiparametric high-dimensional immune monitoring showed that combined treatment of immunocompetent mice bearing syngeneic CT-26 tumors resulted in potent antitumor activity and robust activation of adaptive immune responses, which included intratumor dendritic, NK, and T-cell infiltration and activation and depletion of disease-exacerbating immunosuppressive cell subsets. TAMs and Tregs are frequently associated with tumor-induced tolerance, notably, their capacity to produce immunosuppressive cytokines. We observed a strong negative correlation between the frequency of Tregs and activated T cells, suggesting that the depletion of TAMs could favor CTL activation. In addition, combined therapy did not reduce tumor-infiltrating Treg cells but transformed them into an effector phenotype that expresses a Th1 signature and cooperates with CD8 T cells to kill CT-26 tumor cells through TCR-MHC engagement-dependent mechanisms. These immune responses are likely enhanced by the induced expression of type I IFN gene signatures, which are known to elicit chemotherapy-dependent antitumor immunity through several mechanisms, including promoting cross-priming, supporting cytotoxic T lymphocyte and immune effector functions, and releasing proinflammatory cytokines. Both drugs induced immunosuppressive cell depletion and, consequently, the activation of CD8+ T cells. Mechanistically, T-cell-derived IFN-γ increased the susceptibility of tumor cells to T-cell recognition and elimination via the upregulation of MHC.

In the recent past, TPI was used in clinical trials in combination with checkpoint blockers, viz., bevacizumab and cetuximab, in patients with colorectal cancers (36, 52). However, these attempts were performed using pMMR-MSI-L type CRC. Our data provide strong justification for exploring the potential application of TPI in combination with TLR7-activated DC vaccines to enhance immune responses and cure pMMR-MSI-L-type CRC. The research presented here is innovative and relevant to public health because the combination therapy of TYMP inhibition and imiquimod-activated DC delivers an inventive approach that is universally suitable from patient to patient. This strategy not only establishes a synergistic combination of chemotherapy and immunotherapies in a preclinical model but also has a direct translational impact on one of the deadliest cancers worldwide. The implications of our results may improve patients’ quality of life and provide survival advantages over the best current surgical and chemotherapeutic strategies for this disease.

## Data availability statement

The original contributions presented in the study are included in the article/**Supplementary Material**. Further inquiries can be directed to the corresponding author.

## Ethics statement

This study was reviewed and approved by Dissection Monitoring Committee (DMC) at the University of Burdwan (BU-DMC/2016/01/02).

## Author contributions

AP and SH performed the experiments and analyzed the data. SD and IM performed the histopathological analyses. SH conceived, planned, analyzed, and wrote the manuscript. All authors contributed to the article and approved the submitted version.

## Funding

This study was supported by DST-SERB, GOI as a research grant awarded to SH [CRG/2019/001296] and [EEQ/2021/0594]. AP was supported by the CSIR, India (09/025(0243)/2018-EMR-I) Junior and Senior Research Fellowships.

## Acknowledgments

We thank Bratati Pal and Abhinandan Rej for their assistance with the animal handling. The authors thank Dr. Sankar Bhattacharya, SKBU for flow cytometry (CytoFLEX, Beckman Coulter) and BD Biosciences for multicolor flow cytometry and high-throughput flow cytometry facility and assistance.

## Conflict of interest

The authors declare that the research was conducted in the absence of any commercial or financial relationships that could be construed as a potential conflict of interest.

## Publisher’s note

All claims expressed in this article are solely those of the authors and do not necessarily represent those of their affiliated organizations, or those of the publisher, the editors and the reviewers. Any product that may be evaluated in this article, or claim that may be made by its manufacturer, is not guaranteed or endorsed by the publisher.

## Abbreviation

CRC, Colorectal cancer; 5Fu, 5-fluorouracil; DC, dendritic cell; IMQDC-Ag, imiquimod-activated whole tumor lysate-pulsed DC; IMQ-DC, imiquimod-stimulated DC; ICBs, immune checkpoint blockers; ICD, immunological cell death; MDSC, myeloid-derived suppressor cell; MAbs, monoclonal antibodies; NK, natural killer; PD-ECGF, platelet-derived endothelial cell growth factor; tSNE, t-distributed stochastic neighbor embedding; TYMP, thymidine phosphorylase; TPI, tipiracil hydrochloride; TLRs, toll-like receptors; TAM, tumor-associated macrophage; TILs, tumorinfiltrating lymphocytes; TME, tumor microenvironment; VEGF, vascular endothelial growth factor.
