# Supplementary material for: Targeting thymidine phosphorylase alleviates resistance to dendritic cell immunotherapy in colorectal cancer and promotes antitumor immunity
Source: Front Immunol. 2022 Aug 24;13:988071. doi: 10.3389/fimmu.2022.988071 (PMC9449540; doi:10.3389/fimmu.2022.988071)
Supplement: Supplementary file 1 [file DataSheet_1.pdf]

Supplementary Datasets

1. Supplementary Figures

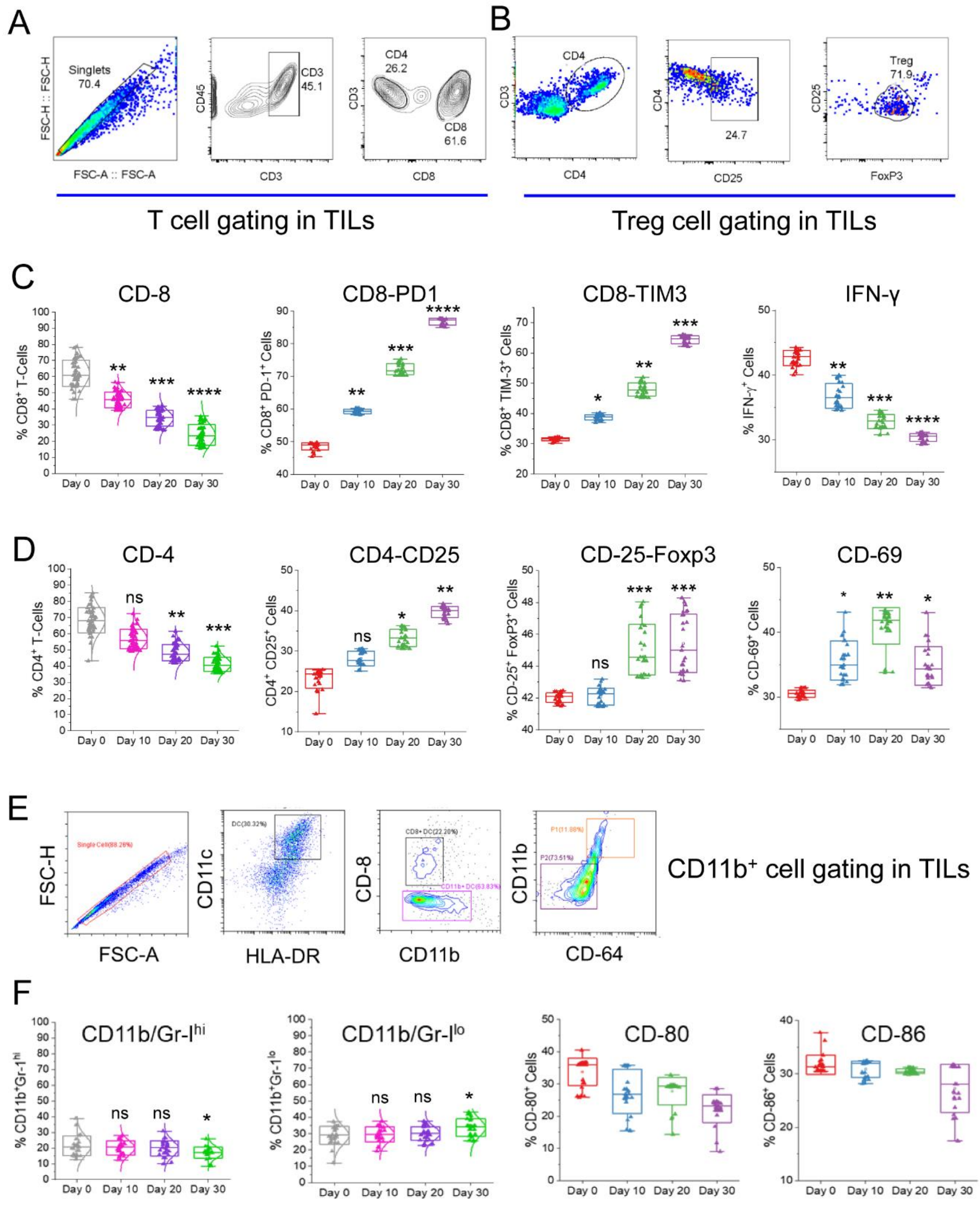

FIGURE S1: (Associated with Fig 1) CD8 T-cell subset skewing and activation patterns in CT26 CRC tumors are potential links to poor prognosis and survival. Gating strategy to identify CD4^{+} and CD8^{+} T cells (**A**) and Treg cells (**B**) in TILs. Percentage of nonnaïve CD8- and CD8-gated PD1-, TIM3-, and IFN-γ-expressing cells (**C**). CD4- and CD4-gated

FoxP3-, CD25- and CD69-expressing cells (**D**). Gating strategy to identify CD11b+/Gr-I^{hi} or ^{lo} cells in TILs (**E**). Percentage of tumor-associated macrophages (TAMs), myeloid-derived suppressor cells (MDSCs) and CD80- and CD86-expressing TILs (**F**). Boxes represent interquartile ranges (IQRs). Each dot represents an individual count, and color represents different days of sample collection. Significance was determined by an unpaired Wilcoxon test with BH correction: *P < 0.05, **P < 0.01, ***P < 0.001, and ****P < 0.0001.

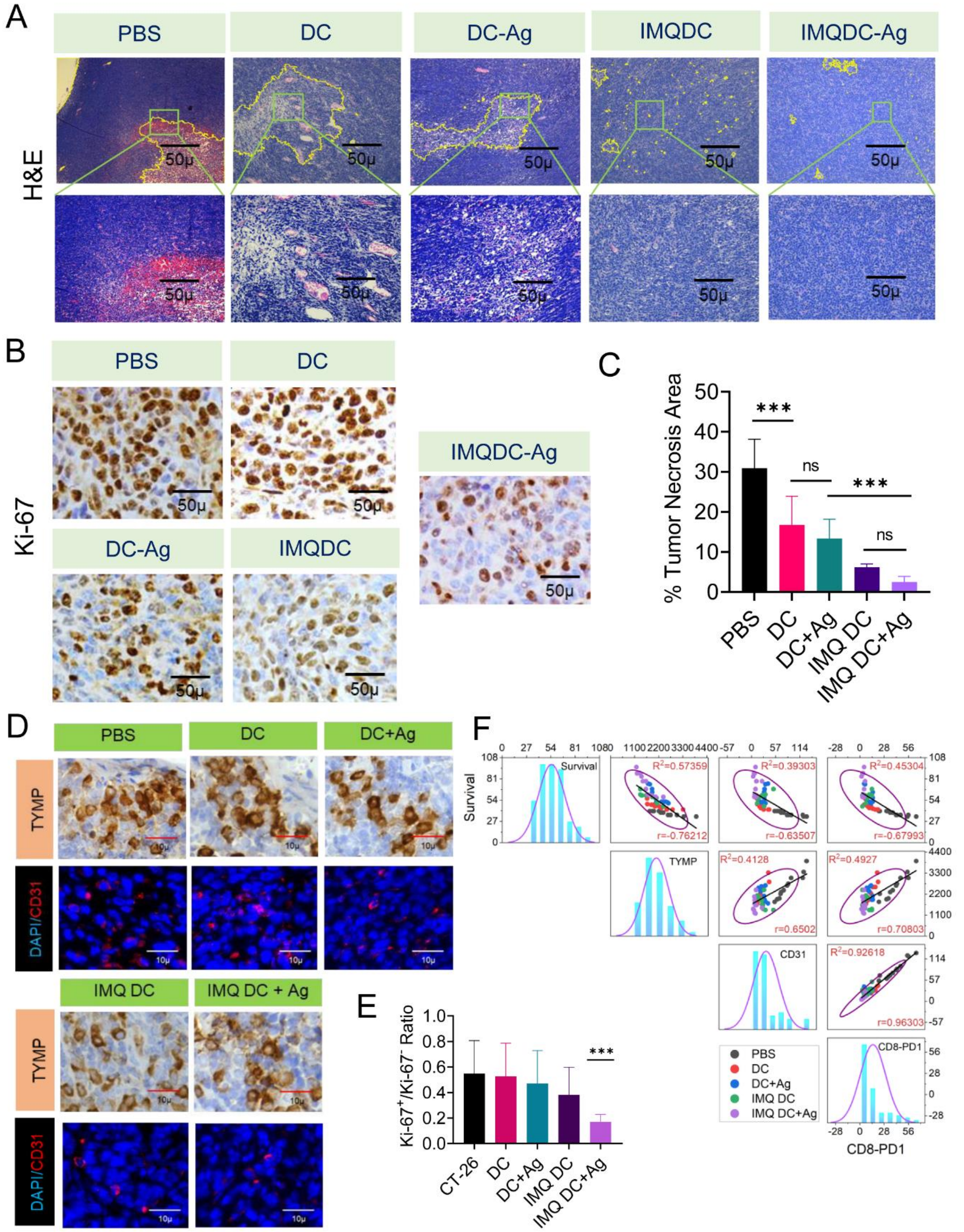

FIGURE S2: (Associated with Fig 2) Histopathological and immunohistochemical analysis of tumors revealed that increased TYMP expression in CRC tumors restrained dendritic cell immunotherapy efficacy by inducing T-cell exhaustion. (A) Histopathology of CT26 colon cancer cell line-induced tumors in mice. Representative H&E-stained tumor sections from PBS = untreated control, DC = BMDC treated, DC+Ag= whole tumor antigen-pulsed BMDCs, IMQDC = imiquimod-activated BMDCs, IMQDC-Ag = IMQDCs pulsed with

tumor antigen. Tumors were resected at day 30 posttreatment. The tumors untreated and treated with DC and DC-Ag had higher mean necrosis percentages than the tumors in the other groups. The insets illustrate numerous mitotic features, grades of necrotic zones (border by a yellow line) and numerous apoptotic bodies. **(B)** Representative tumor tissue sections from three mice showing immunostaining for Ki-67. The bar indicates 50 μm. **(C)** Comparison of the percentage of necrosis by H&E staining was performed using the MIPAR software package. **(D)** Representative immunohistochemical localization of TYMP and immunofluorescence localization of CD31 in CRC tumors at day 30. **(E)** The $Ki67^{+}/Ki67^{-}$ ratio differed throughout the treatment groups. The untreated control group was characterized by a high $Ki67^{+}/Ki67^{-}$ ratio, and the other group showed varying $Ki67^{+}/Ki67^{-}$ ratios. **(F)** Multivariate scatter plot matrix correlation analysis showing histograms, absolute correlations and correlation coefficients (r) for the relationship between TYMP, CD31, CD8-PD1 cells and survival over time. Multivariate analysis predicted that TYMP overexpression in CRC tumors restrains dendritic cell immunotherapy efficacy by inducing T-cell exhaustion. Significance was determined by an unpaired Wilcoxon test with BH correction: *P < 0.05, **P < 0.01, ***P < 0.001, and ****P < 0.0001.

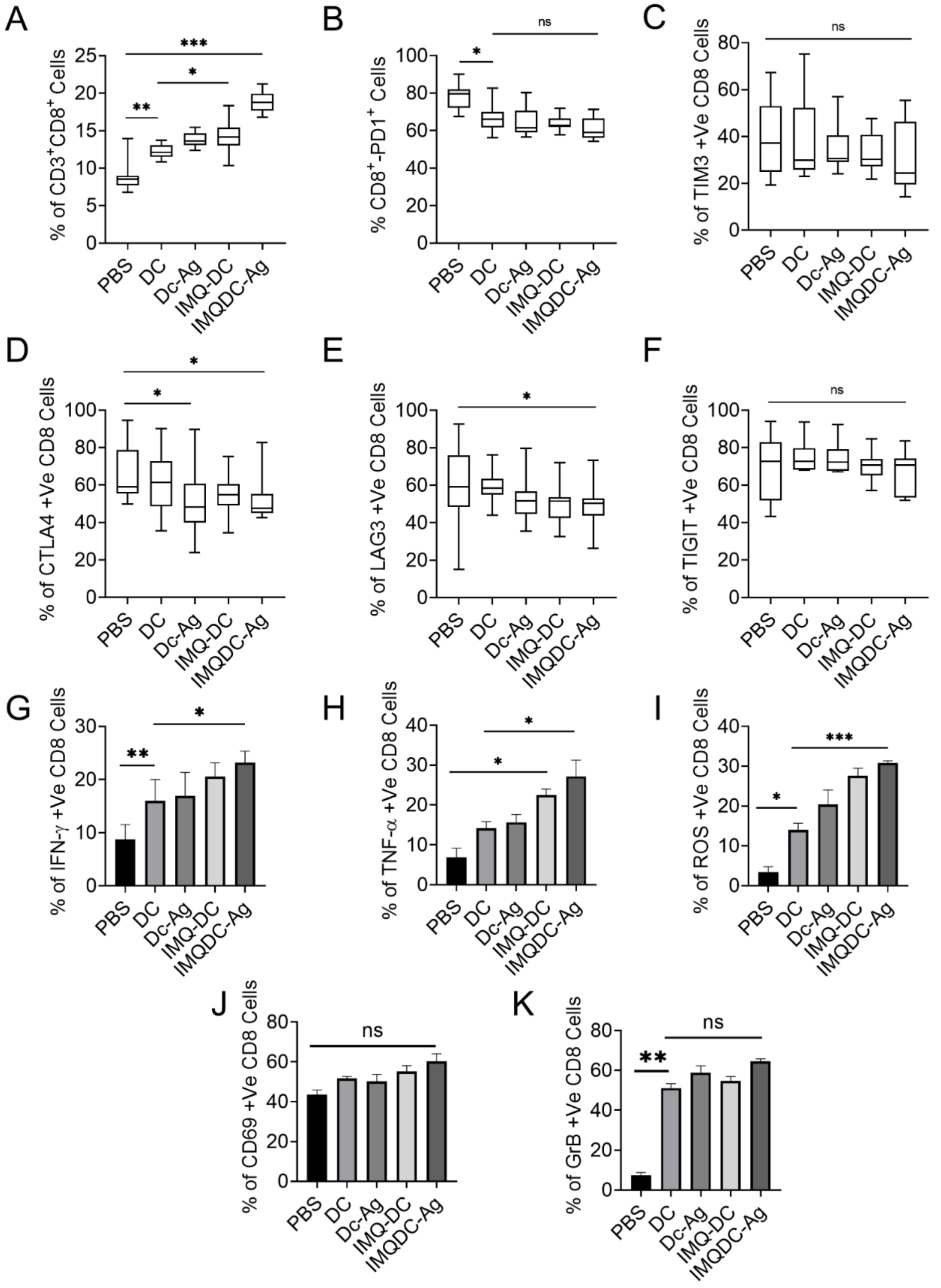

FIGURE S3: (Associated with Fig 2h-k) Percentage of CD3^{+}CD8^{+} (**A**) and CD8 gated PD1 (**B**), TIM3 (**C**), LAG3 (**D**), TIGIT (**E**), CTLA4 (**F**) and IFN-γ (**G**), TNF (**H**) ROS (**I**) GrB (**J**) CD69 (**K**) expressing cells. Boxes represent interquartile ranges (IQRs). Each dot represents an individual count, and color represents different days of sample collection. Significance was determined by an unpaired Wilcoxon test with BH correction: *P < 0.05, **P < 0.01, ***P < 0.001, and ****P < 0.0001.

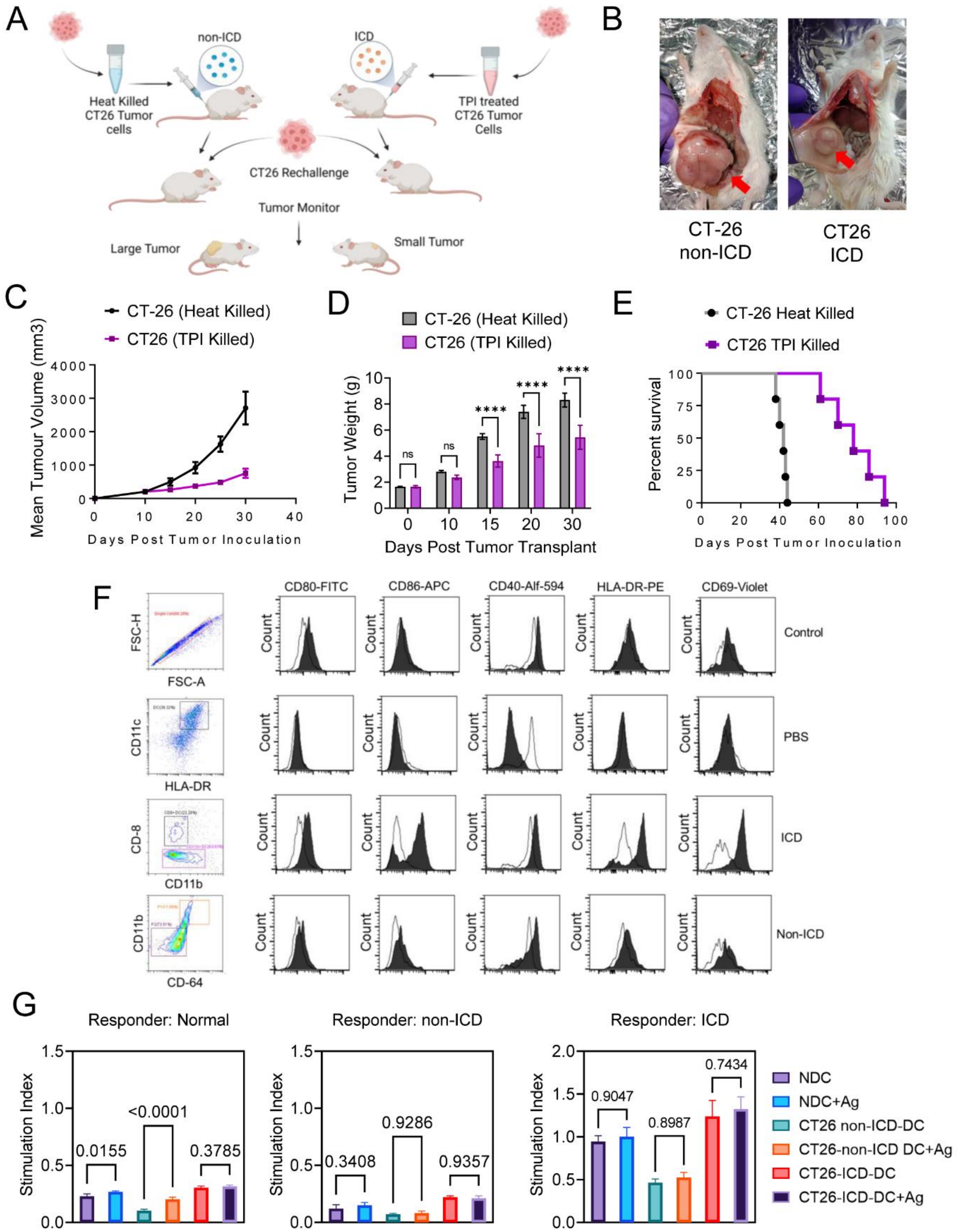

FIGURE S4: (Associated with Fig 3) Assays for the evaluation of immunogenic cell death in vivo. (**A**) Vaccination assays. CT26 murine cancer cells were exposed to TPI in vitro to a putative inducer of immunogenic cell death (ICD). Heat-killed CT26 cells (negative control) were washed, resuspended in PBS, and eventually injected s.c. into one flank (vaccination site) of immunocompetent syngeneic mice (n=5). One week later, mice were challenged with living CT26 cells, which were inoculated s.c. into the contralateral flank (challenge site). (**B-E**)

Tumor incidence and growth are routinely monitored at both injection sites over a 2–3-month period. (**F**) FACS analysis and quantification of CD11c+/Class II DCs in the spleens of untreated CT26 or CT26 mice with the indicated treatment. DCs from healthy animals were used as controls. Representative of one experiment out of three performed is shown. (**G**) Antigen-specific DC-mediated immune response. Significance was determined by an unpaired Wilcoxon test with BH correction: *$P < 0.05$, **$P < 0.01$, ***$P < 0.001$, and ****$P < 0.0001$.

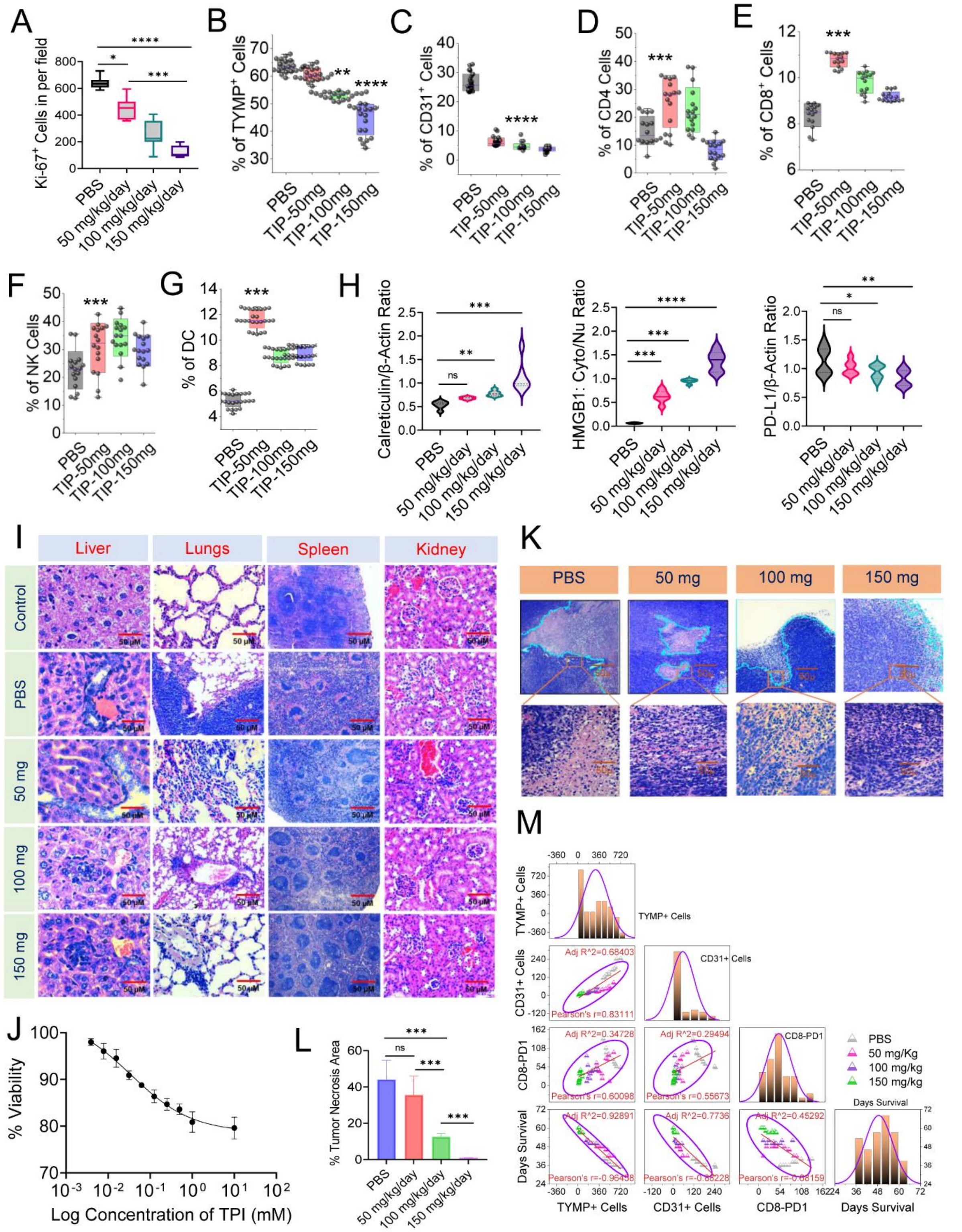

FIGURE S5: (Associated with Fig 4) TPI monotherapy induces ICD, and ICD-associated DAMPs mediate dendritic cell (DC) maturation, reduce tumor metastasis in distal organs and increase the percentage of tumor necrosis. (A) Quantitative analysis of Ki-67-positive cells per high-power field. Ki-67-positive cells showed a brown nuclear pattern (**FIGURE 4g**). Data represent the mean ± SE of n = 3 or 4 animals per group. Percentages of TYMPs (**B**), $CD31^+$ (**C**), $CD4^+$ (**D**), $CD8^+$ (**E**), NK cells (**F**) and DCs (**G**) from the FlowSOM cluster

(**FIGURE 4i**). Boxes represent interquartile ranges (IQRs). **(H)** Normalized ratio of CRT, HMGB1 and PDL1 against β-Actin and H3 loading controls for the cytoplasmic and nuclear fractions, respectively. **(I)** Histopathological analysis of the liver, lung, spleen and kidney of CT26 untreated, 50, 100 or 150 mg kg-1 body TPI-treated mice compared with healthy control magnification 400×. Different grades of metastasis in the liver and lung of untreated or treated groups were observed. (**J**) Assessment of the toxic effect of TPI on IMQDC. (**K**) Representative H&E-stained tumor tissue sections from untreated and treated mice with TPI of different grades of necrosis. (**L**) Comparison of the percentage of necrosis by H&E staining was performed using the MIPAR software package. (**M**) Multivariate scatter plot matrix correlation analysis showing histograms, absolute correlations and correlation coefficients (r) for the relationship between TYMP, CD31, CD8-PD1 cells and survival rate over time among different treatment groups. Boxes represent interquartile ranges (IQRs). Each dot represents an individual count, and color represents different days of sample collection. Significance was determined by an unpaired Wilcoxon test with BH correction: *$P < 0.05$, **$P < 0.01$, ***$P < 0.001$, and ****$P < 0.0001$.

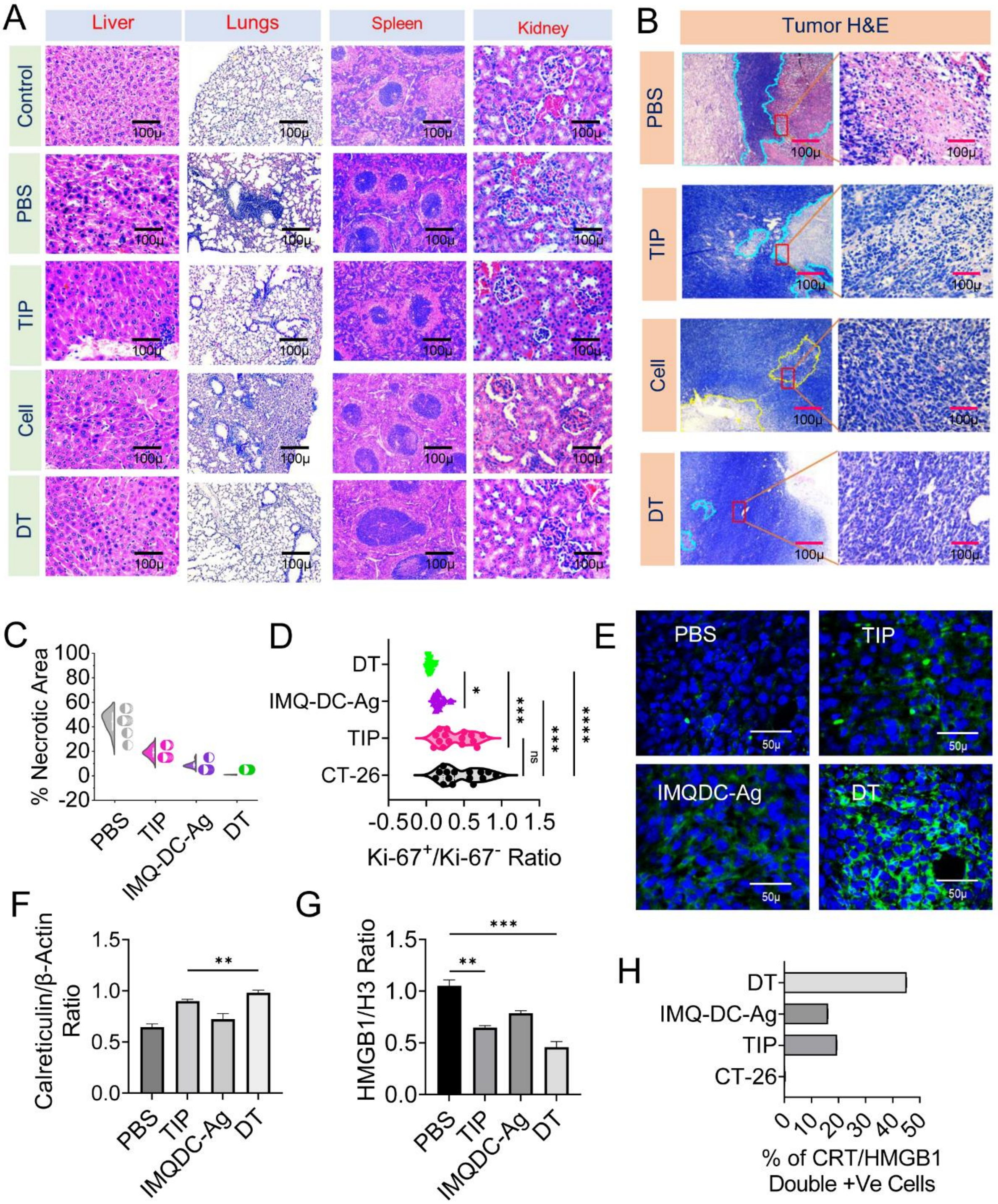

FIGURE S6: (associated with Fig 5) Histopathological analysis of tumor invasion and ablation in untreated and vaccinated CT-26 tumor-bearing mice. (A) Histopathological analysis of the liver, lung, spleen and kidney of CT26 untreated, TPI- or IMQDC-Ag only or TPI+IMQDC-Ag-treated mice compared with healthy control magnification 400×. (**B**) Tumor architecture in untreated CT26 cells and mice treated with either TPI or IMQDC-Ag only or TPI+IMQDC-Ag was analyzed by H&E-stained histopathological sections. The tumors treated with TPI+IMQDC-Ag had lower mean necrosis percentages than the tumors in the other groups. The insets illustrate numerous mitotic features, grades of necrotic zones (bordered by

yellow and blue lines) and numerous apoptotic bodies. (**C**) Comparison of the percentage of necrosis by H&E staining was performed using the MIPAR software package. (**D**) The Ki67+/Ki67- ratio differed throughout the treatment groups. The untreated control group was characterized by a high Ki67+/Ki67- ratio, and the other group showed varying Ki67+/Ki67- ratios. (**E**) Immunofluorescence staining of CRT in tumor sections of the indicated treatment groups. (**F-G**) Normalized ratio of CRT & HMGB1 against β-Actin and H3 loading controls for the cytoplasmic and nuclear fractions, respectively. (**H**) Bar diagram representing the % of CRT/HMGB1-positive cells from the flow cytometric analysis of CRT and HMGB1 in tumor tissue presented in Fig 5j.

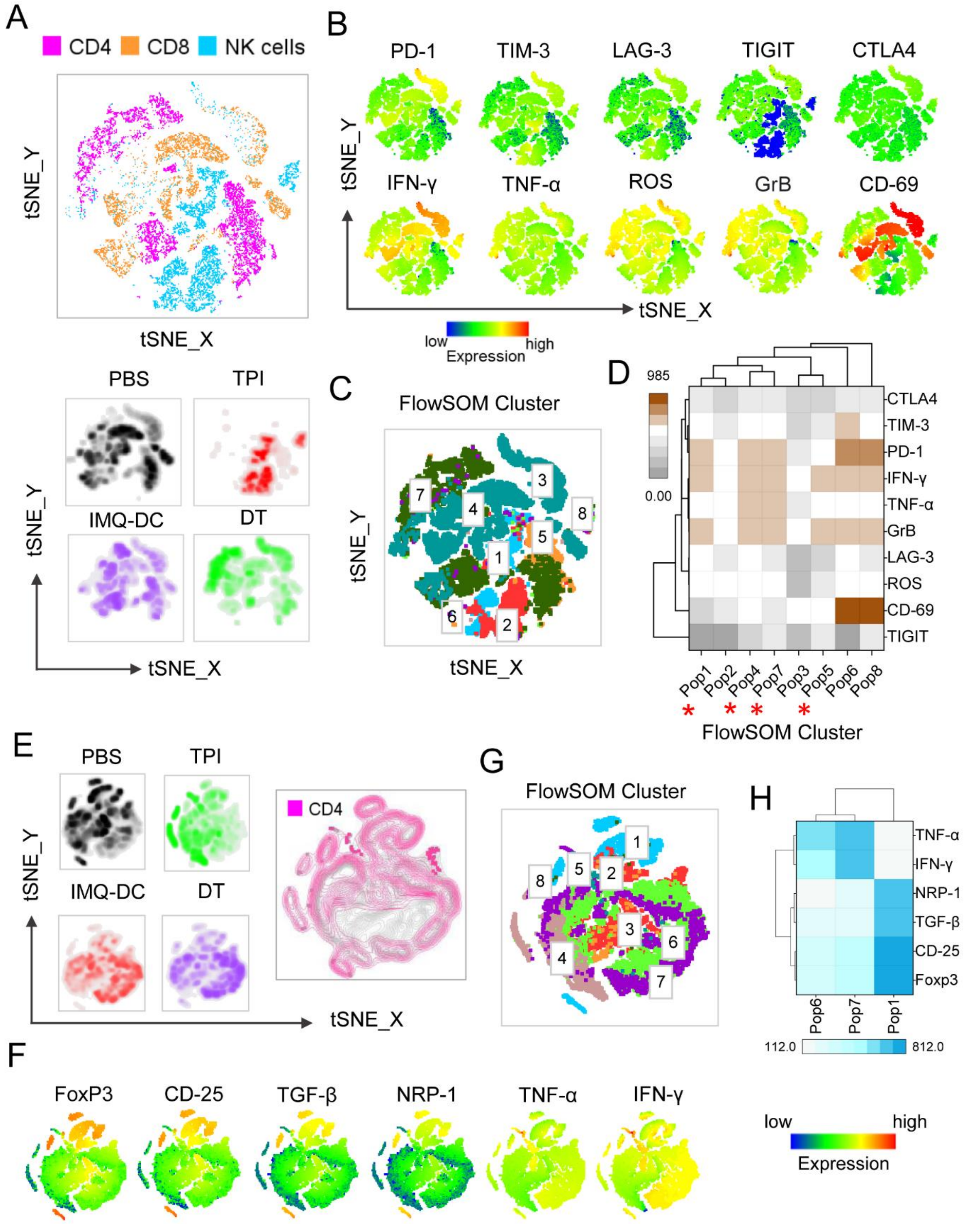

FIGURE S7: (Associated with Fig 6) Deep profiling of tumor infiltrating T-cell populations reveals robust CD8 effector populations and other T-cell phenotype alterations in chemoimmunotherapy. (A) (top) Global tSNE projection of CD4^{+} T cells (pink dots), CD8^{+} T cells (yellow dots), and NKs (blue dots) for all samples of the DT group pooled, concatenated, and overlaid. (bottom) Islands within the tSNE space represent major populations within the indicated treatment group. **(B)** tSNE maps showing the expression level

and the distribution of each indicated marker. The color scale indicates the relative expression levels. **(C)** tSNE projection of nonnaïve $CD8^+$ T-cell clusters identified by FlowSOM clustering. **(D)** Heatmap showing the MFI (column-scaled z scores) in the indicated clusters. The color scale indicates the relative expression levels. * Indicates clusters 4, 5 & 7, which are increased $CD8^+$ effector populations. **(E)** (left) $CD4^+$ T-cell islands within the tSNE space represent major populations within the indicated treatment group. (right) Global tSNE projection of CD4 T cells from all treatment groups concatenated and overlaid. **(F)** tSNE maps showing the expression level and the distribution of each indicated marker. The color scale indicates the relative expression levels. **(G)** tSNE projection of CD4 T-cell clusters identified by FlowSOM clustering. **(H)** MFI as indicated (column-scaled z scores) for selected populations. tSNE and FlowSOM analyses were performed by FlowJo v10.8.0. DCs, NK cells, nonnaïve CD4 T cells, and nonnaïve CD8 T cells were analyzed separately. tSNE analysis was performed using equal sampling of 1000 cells from each FCS file, with 5000 iterations, a perplexity of 30, and a theta of 0.5.

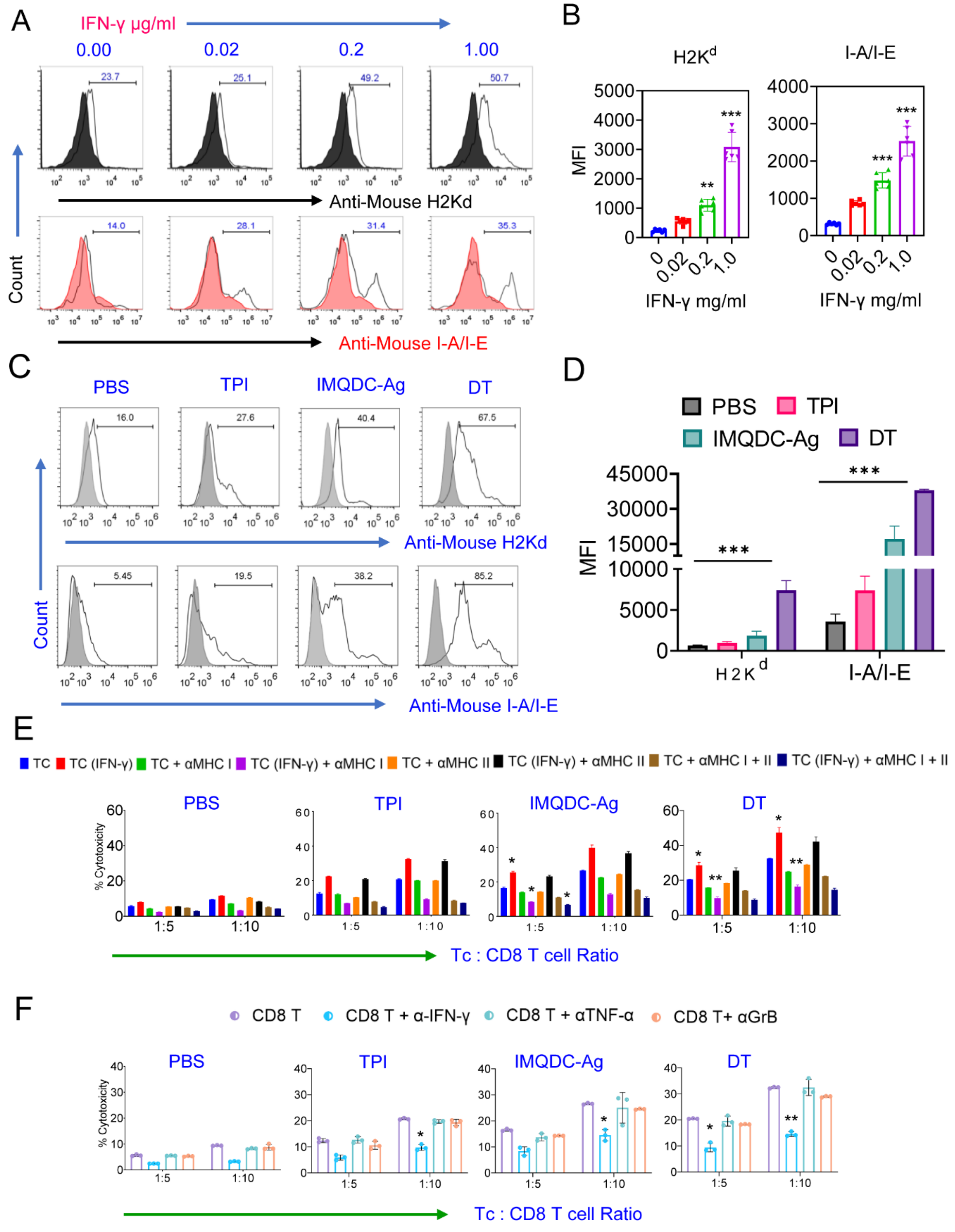

FIGURE S8: MHC-dependent recognition is required for the antitumor activity of reactivated CD8 T cells. Flow cytometric measurement of the expression of MHC class I ($H2K^d$) and MHC class II (I-A/I-E) on CT26 cells after treatment with IFNγ in vitro. Each condition was tested as 3 biological replicates (**A-B**). Flow cytometric measurement of the expression of MHC class I (H2Kd) and MHC class II (I-A/I-E) on CT26 tumors among different indicated treatment groups in vivo (**C-D**). Tumor cells (CT26, $5x10^3$ cells) were

cocultured for 48 hours with CD8 T cells from the indicated treatment groups at a 1:5 or 1:10 ratio. The percent cytotoxicity was measured using commercial cytotoxicity assay kits. Tumor cells (TCs) were either pretreated or untreated with 1.0 µg/ml IFN-γ before the addition of CD8 T cells. T-cell-mediated TC cytotoxicity was measured under treatment with i) IgG, ii) αMHCI, iii) αMHCII, and iv) αMHCI&II (**E**). Tumor cells were cocultured for 72 hours with CD8 T cells harvested from the indicated treatment groups at a 1:5 or 1:10 ratio in a Transwell plate. Tumor cell cytotoxicity was measured under treatment with i) IgG, ii) αIFNγ iii) αTNFα, and iv) αGzmB (**F**). Data presented are the mean ± SD. All p values were calculated using one-way ANOVA and corrected for multiple comparisons using Tukey's adjustment. NS no significance; *$P < 0.05$, **$P < 0.01$, ***$P < 0.001$, and ****$P < 0.0001$.

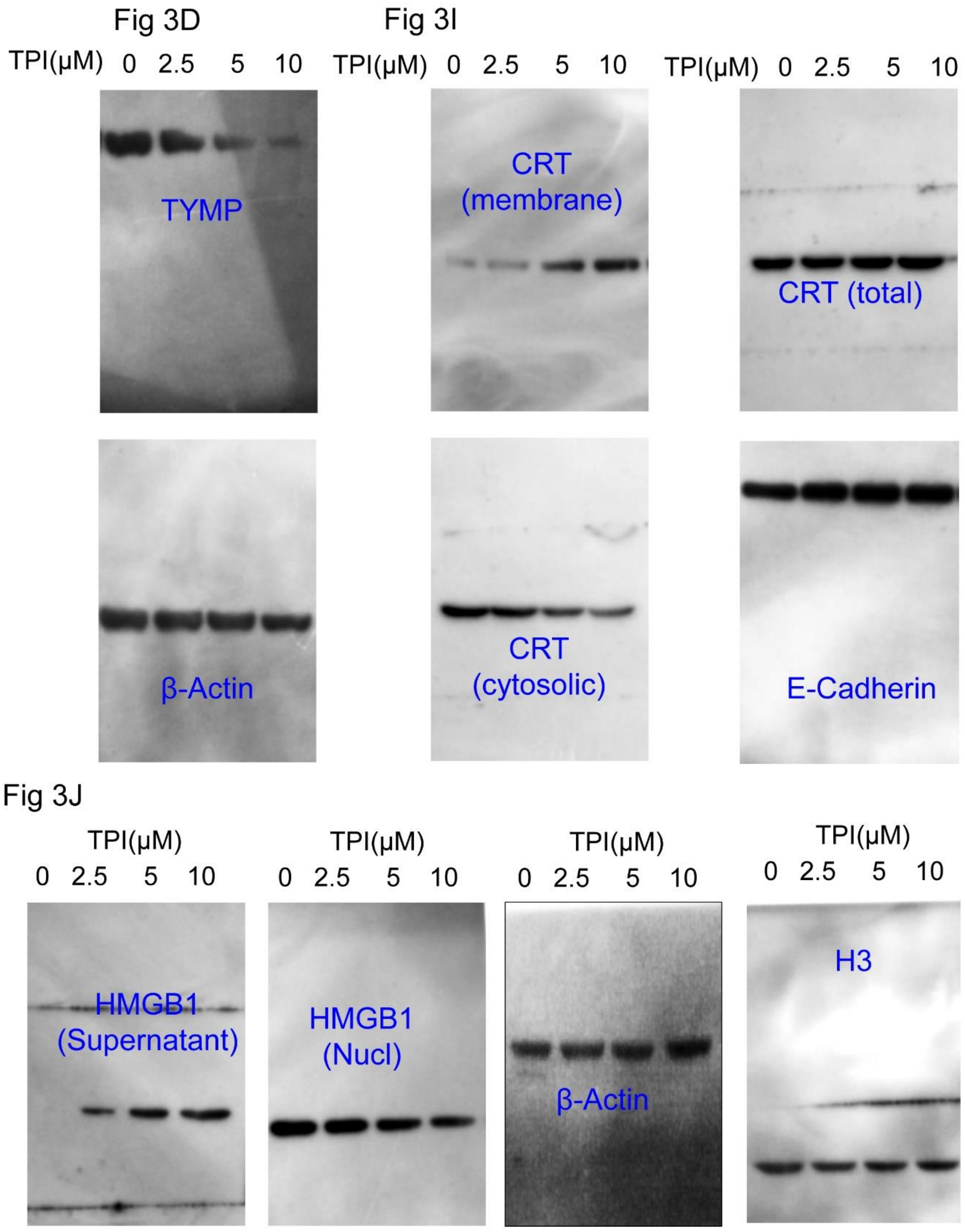

FIGURE S9: Representative uncropped western blot images associated with FIGURE 3

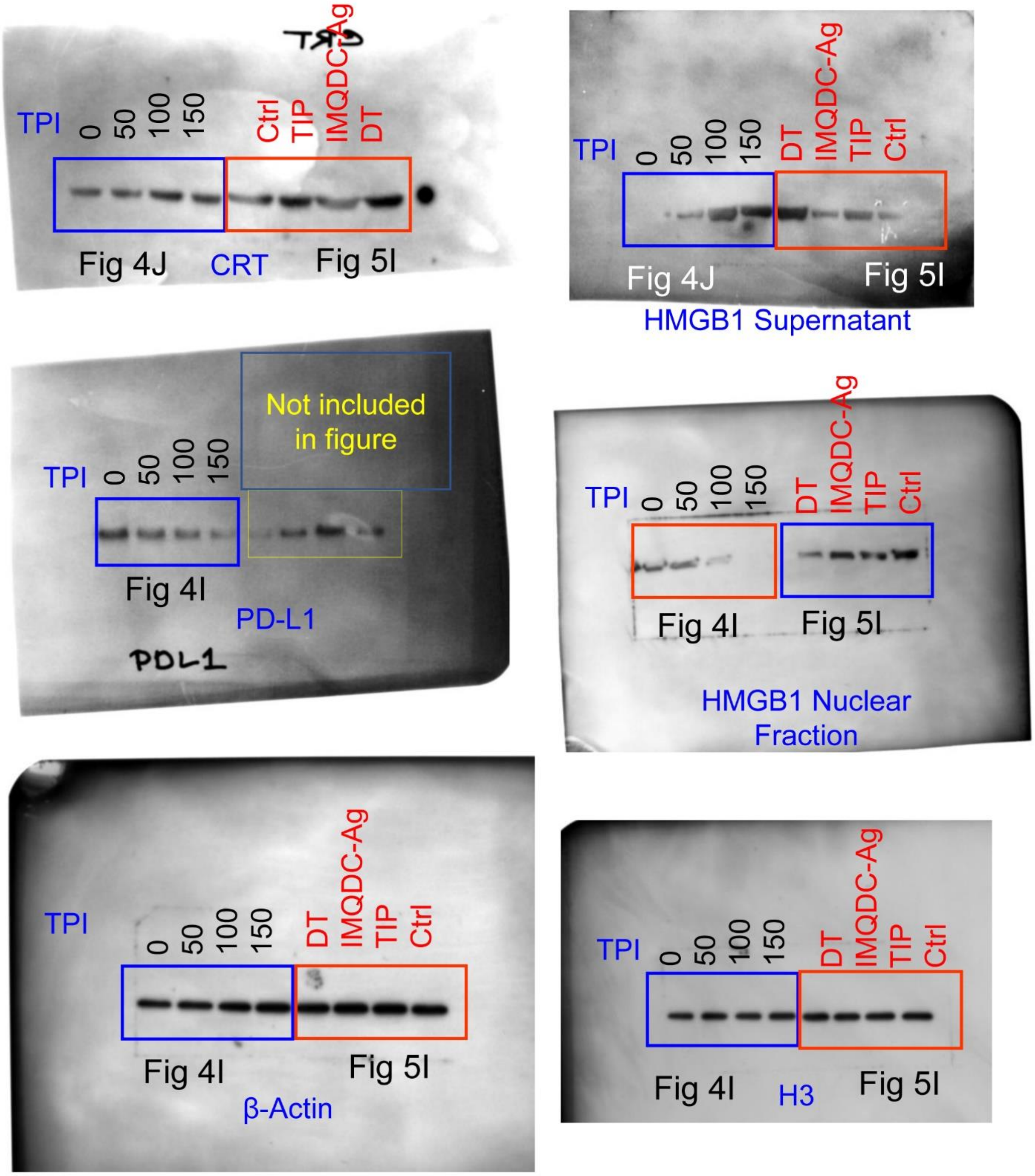

FIGURE S10: Representative Uncropped western blot images associated with FIGURE 4 & FIGURE 5

2. Supplementary Methods

Cells and Animals

CT-26, MC38, HCT-116, HCT-15, Caco-2, 4T1, MCF-7 and C127I tumor cells were acquired as previously described (1, 2) and were not further authenticated. All cell lines were regularly tested for mycoplasma contamination every 4 weeks. Cells were thawed at early passages and cultured for up to 32 weeks in total. Six- to eight-week-old male Balb/c mice (from Charles River Laboratories) were ordered from Hylasco Biotechnology, Hyderabad. All mice were maintained under SPF housing with a maximum of six mice per cage. Food and water were available ad libitum. The health status of the mice was routinely checked by veterinary staff. Animals were sacrificed at the indicated time by injecting a high dose of ketamine/xylazine, and blood samples and tissues were collected. Animal studies were conducted under the approval of the Institutional Dissection Monitoring Committee (DMC) at the University of Burdwan.

BMDC culture and stimulation

BMDCs were isolated from the femurs of Balb/c mice as described (3), and some of the BMDCs were incubated with GMCSF (100 ng/mL), imiquimod (200 pg/mL) or LPS (5 mg/mL) for 16 h to induce maturation and differentiation. Mature BMDCs were pulsed with whole tumor cell lysates (Ag) (10 mg/mL) as previously described (3).

In vitro antitumor efficacy and apoptosis detection

The thymidine phosphorylase inhibitor tipiracil hydrochloride (TPI) was obtained from Selleck Chemicals (S3731), Houston, TX. To evaluate the effect of TPI (CT-26, MC38) on cell proliferation, cells were plated in 96-well plates at 10×10^3 cells/well followed by treatment with increasing concentrations of TPI (0, 0.25, 0.5, 1, 2.5, 5, 10, 25, 50, 100 µM) for 48 hours. CellTiter 96® Non-Radioactive Cell Proliferation Assay (MTT) (Promega, Fitchburg, WI, USA) was used to quantify cell proliferation according to the manufacturer's protocol. IC_{50} values of TPI were determined by curve fitting using nonlinear regression (GraphPad Prism 9.0), which reports median-effect plots, the median dose (Dm, IC_{50}) and the correlation coefficient I. The 95% confidence intervals of the IC_{50} were determined using GraphPad Prism 9 software. To assess the TPI-mediated apoptosis of CT-26 cells, Annexin-FITC/PI staining was performed as described earlier (2).

DC functional assays

Mouse BMDCs or human peripheral blood DCs were cultured in 96-well plates with medium alone (naïve DCs) or in the presence of GMCSF (1000 U/mL) (GMCSF DCs), imiquimod (200 pg/mL) (IMQ DCs) or LPS (5 µg/mL) (LPS DCs). After 6 hours, the cells were washed (×3) and added to wells containing murine (CT-26, MC38, 4T1, C127i) and human (HCT116, HCT-15, HepG2, Caco2, MCF-7) cells for mouse and human DCs, respectively, at a 1:1 E:T ratio. The tumor cells were pretreated with or without 2.5 µM TPI. To assess DC-mediated growth inhibition (48 hours), an MTT assay was performed as described earlier (3).

Transplantable CT-26 tumors

Tumor formation was induced by subcutaneous injection of 5×10^4 CT-26 cells in 100 µL of serum-free RPMI into the hindquarters of Balb/c male mice. Mice were randomized to experimental groups when subcutaneous tumors reached 50 mm^3. Ten days after tumor cell implantation, when the average tumor size reached 90 mm^{3,} tumor monitoring was initiated (noted as Day 0). Tumor volume was calculated as (length × width × height)/2.

Antitumor efficacy against s.c. CT-26 tumors in Balb/c mice

Male Balb/c mice (6-8 weeks old from Charles River Laboratories) were injected subcutaneously in the right flank with CT-26 cells (5 x 10^4 cells in 0.1 mL). Mice were randomized into groups, and tipiracil hydrochloride (TPI) treatment began when the average tumor volume was ~80-100 mm^3 on day 10. For the control group, vehicle (0.5% HPMC solution, 10 mL/kg) was orally administered. TPI at the indicated (50, 100 & 150 mg/kg/day) doses was orally administered twice a day followed by 1 drug-free day for 4 days. After a gap of 6 days, TPI was again administered orally once a day followed by 1 drug-free day for 8 days.

In different experiments, CT-26 tumor-bearing mice treated with 8 intraperitoneal (i.p.) injections of whole CT-26 tumor lysate-pulsed DCs or imiquimod-activated CT-26 whole tumor-pulsed DCs (1×10^6 cells/mouse) either alone or in addition to TPI (50 mg/kg body weight) by oral gavage for 22 days. Altogether, six TPI doses and 8 DC doses were given at intervals of 24 hours. Therapeutic efficacy was determined by calculating the tumor burden for all mice over the course of the study by taking the area under the curve (AUC) of tumor volume over time. Adjusted AUC values for all mice in each group were analyzed by nonparametric analysis of variance (ANOVA on the ranks). Furthermore, the incidence, magnitude, and durability of regression responses were also evaluated. An animal with a complete regression response (six consecutive measurements) at the termination of a study was classified as a tumor-free survivor. Survival was analyzed by the Kaplan–Meier method. Groups of mice were compared by log-rank test.

Histology, Immunohistochemistry and Immunofluorescence

Unless otherwise specified, all histology, immunohistochemistry (IHC), immunofluorescence (IF) analysis and TUNEL assays were performed in formalin-fixed, paraffin-embedded tissue sections as described earlier (4, 5), and images of histology and IHC were captured by a Nikon 80i research microscope (100× and 400×). All IF images were observed and captured with a DMi8 fluorescence microscope (Leica, Germany). Acquired images were analyzed using ImagePro 10 (Media Cybernetics) software. The captured IHC images were further analyzed by MIPAR image analysis software for quantitative estimation. The antibodies used for IHC and IF staining is provided in **Supplemental Table S4**.

Western Blot

For western blotting analysis, RIPA lysis buffer (10 mM Tris-HCl [pH 7.4], 50 mM NaCl, 0.05% SDS, 10 mM Na2-EDTA, 0.5% Triton X-100, 0.5% sodium deoxycholate, supplemented with protease and phosphatase inhibitors [Roche]) was used for total protein extraction. Cytoplasmic proteins and nuclear proteins were separated by a Cytoplasmic and Nuclear Extraction Kit from Genetix Biotech Asis Pvt. Ltd. India (GX-6010AR). The membrane proteins were isolated by a Mem-PER™ plus membrane

protein extraction kit (89842) from Thermo Scientific. The protein concentration of each sample was measured by Bradford kits (Amresco, USA). After SDS–PAGE, the proteins in the cell lysates were separated and then transferred to a PVDF membrane. The membrane was blocked with 5% nonfat powdered milk or 5% BSA in TBST buffer for 1 h and incubated with diluted primary antibodies (listed in **Supplemental Table S5**) at 4 °C overnight. After washing with TBST buffer, the membrane was incubated with an HRP-coupled secondary antibody at room temperature for 1 h. The membrane was visualized with SuperSignal™ West Pico PLUS chemiluminescent substrate (34580) from Thermo Scientific. The gray value ratio of the target band to the internal reference analyzed by myImageanalyis software from Thermo Scientific represents the relative protein expression. For analysis of released HMGB1, the culture supernatant was concentrated using an Amicon Ultra centrifugal filtration unit (UFC901024, Merck-USA).

Measurement of ATP release

Measurement of ATP release in untreated or TPI-treated CT-26 cells was measured using the commercial ATP Bioluminescence Assay Kit HS II (11699709001) from Roche, USA**,** according to the manufacturer's instructions (5)**.**

Tumor-infiltrating lymphocyte analysis for in vivo antitumor immune response

The harvested tumors from the indicated treatment groups were explanted and cut into small pieces. Then, the small pieces were immersed in 5 mL collagenase IV (1 mg mL^{-1}) with 0.2 mg mL^{-1} DNase I for 1 h at 37 °C. Suspensions were filtered through a cell strainer, and after gating on $CD45^+$ cells, single cells were stained with fluorescently labeled antibodies ($CD4^+CD25^+Foxp3^+$ Tregs, $CD4^-CD8^+$ T cells, $CD8^+$IFN-γ^+, $CD8^+PD1^+TIM3^+LAG3^+$ T cells, $CD11c^+$/Class II^+/$CD80^+$/$CD86^+$/$CD40^+$ DCs, CD8+/CD44+/CD62 L+ memory T cells). Fluorescence-labeled antibodies were diluted according to the manufacturer's instructions (Supplementary Table S2).

Flow Cytometry

Approximately 1×10^5 to 1×10^6 freshly isolated tumor infiltrating lymphocytes (TILs) were used per individual animal per stain. See Table S2 for buffer and antibody panel information. TILs were stained with live/dead mix (100 µl, 10 min, RT), washed with fluorescence-activated cell sorting (FACS) buffer, and spun down (700 g, 5 min, RT). TILs were incubated with Fc block (TruStain FcX, BioLegend, catalog no. 101319, RT, 10 min) before a second wash (FACS buffer, 700 g, 5 min, RT). The pellet was resuspended in cell surface receptor staining mix and incubated at 37 °C for 45 min. TILs were washed (FACS buffer, 700 g, 5 min, RT), stained with secondary antibody mix for 30 min at RT and then washed again (FACS buffer, 700 g, 5 min, RT). Samples were fixed and permeabilized by incubating in Fix/Perm buffer (RT, 30 min) and washing in Perm Buffer (1000 g, 5 min, RT). TILs were stained with intracellular mix overnight at 4 °C. The following morning, the samples were washed (Perm Buffer, 800 g, 5 min, RT) and further fixed in 1.2% paraformaldehyde (PFA). Before acquisition, the samples were diluted, and 10,000 events were counted. A live/dead mix was prepared in phosphate-buffered saline (PBS). For the surface receptor and cytokine staining mix, antibodies were diluted in FACS buffer. Intracellular mix was diluted in Perm Buffer. Samples were acquired on a five-laser BD LSRFortessa™. Standardized SPHERO rainbow beads (Spherotech, catalog no. RFP-30-5A) were used to track and adjust photomultiplier tubes over time. UltraComp eBeads

(ThermoFisher, catalog no. 01-2222-42) were used for compensation. Up to 1×10^6 live TILs were acquired per sample.

Cytokine quantification

TILs from animals of different treatment groups were rested overnight at 37 °C in complete RPMI. Flat-bottom 96-well plates were coated with 1 mg/ml anti-CD3 in PBS at 4 °C overnight. The next day, cells were collected and plated at 1×10^5 per well in 100 µl in duplicate. Anti-mouse CD28/CD49d (2 mg/ml) was added to the wells containing plate-bound anti-CD3. TILs were stimulated or left unstimulated for 16 hours and spun down (600 g, 10 min), and the supernatant (85 µl per well) was collected. Cytokine quantification was run according to the manufacturer's instructions using a custom mouse cytokine multiplex-assay panel (LEGENDplex™ MU Th1/Th2 Panel (8-plex), BioLegend, catalog no. 741053). The panel included IL-5, IL-13, IL-2, IL-6, IL-10, IFN-γ, TNF-α, and IL-4, which are collectively secreted by Th1 and Th2 cells. Data acquisition and analysis were performed using a Cytoflex flow cytometer (Beckman). Data generated using LEGENDplex™ kits were analyzed using freely available LEGENDplex™ data analysis software. Protein levels of IL-2, IL-6, IL-10, IL-12p60, IFN-γ, TNF-α, VEGF and TGF-β in serum samples of different treatments were determined by commercial ELISA kits from BioLegend, USA, as listed in Table S3.

Statistics

All statistical analyses were performed using Origin2021b, SAS, R or GraphPad Prism 9. One-way ANOVA and two-way ANOVA were used as indicated to compare continuous outcomes across multiple experimental groups. For all tests, $P < 0.05$ was considered significant. The sample size was not predetermined. Unless otherwise noted, the samples were independent biological replicates. For *in vitro* and *in vivo* studies, analysis of variance and t and chi-squared tests were used as indicated to compare independent groups. Survival functions were estimated by the Kaplan–Meier method and were compared using the log-rank test.

Correlation plots and heatmap visualization

Pairwise correlations between variables were calculated and visualized as a correlogram using the R function corrplot. Spearman's rank correlation coefficient (r) is indicated by the square size and heat scale; significance is indicated by $*P < 0.05$, $**P < 0.01$, and $***P < 0.001$; and a black box indicates a false discovery rate (FDR) < 0.05. Heatmaps were created to visualize variable values using the R function pheatmap or complexheatmap.

High-dimensional data analysis of flow cytometry data

tSNE and FlowSOM analyses were performed by FlowJo v10.8.0, BD Biosciences. DCs, NK cells, nonnaïve $CD4^+$ T cells and $CD8^+$ T cells were analyzed separately. tSNE analysis was performed using equal sampling of 1000 cells from each FCS file, with 5000 iterations, a perplexity of 30, and a theta of 0.5. For nonnaïve $CD4^+$ and $CD8^+$ T cells, the following markers were used: CD45RA, FOXP3, CD25, neuropilin-1, PD1, TIM3, LAG3, TIGIT, IFN-γ, CTLA4, ROS, granzyme B, CD69, CD44, CD62 L, and Class II. The resulting tSNE maps were fed into the FlowSOM clustering algorithm. For each cell subset, a new self-organizing map (SOM) was generated using hierarchical consensus

clustering on the tSNE axes. For each SOM, 7 or 8 clusters were identified for T cells. To group individuals on the basis of the T-cell landscape, pairwise EMD values were calculated on the T-cell tSNE axes using the emdist package in R. The resulting scores were hierarchically clustered using the hclust package in R. For DCs, CD11b, CD11c, CD8, Class II, CD80, CD86, and CD40 and for NK cells, CD3, NKp64, CD49b, and NKG2D markers were used.

Batch effect correction

During sample acquisition in flow cytometry, batch correction was performed to remove potential bias from downstream analysis and reduce variability across batches. A variance stabilizing transform (logit) was applied to each data value prior to recentering the second panel. After batch correction, neither tSNE_X nor tSNE_Y had a statistically significant difference between panels by unpaired Wilcoxon test.

2.1. Supplementary Reference

1. Srivastava P, Hira SK, Srivastava DN, Gupta U, Sen P, Singh RA, et al. Protease-Responsive Targeted Delivery of Doxorubicin from Bilirubin-BSA-Capped Mesoporous Silica Nanoparticles against Colon Cancer. ACS Biomater Sci Eng. 2017;3(12):3376-85.

2. Hira SK, Rej A, Paladhi A, Singh R, Saha J, Mondal I, et al. Galunisertib Drives Treg Fragility and Promotes Dendritic Cell-Mediated Immunity against Experimental Lymphoma. iScience. 2020;23(10):101623.

3. Hira SK, Mondal I, Bhattacharya D, Manna PP. Downregulation of endogenous STAT3 augments tumoricidal activity of interleukin 15 activated dendritic cell against lymphoma and leukemia via TRAIL. Exp Cell Res. 2014;327(2):192-208.

4. Paladhi A, Rej A, Sarkar D, Singh R, Bhattacharyya S, Sarkar PK, et al. Nanoscale Diamond-Based Formulation as an Immunomodulator and Potential Therapeutic for Lymphoma. 2022;13.

5. Kashif M, Paladhi A, Singh R, Bhattacharyya S, Hira SK, Manna PP. Leishmanicidal Activity of an In Silico-Screened Novel Inhibitor against Ascorbate Peroxidase of Leishmania donovani. 2020;64(7):e01766-19.
