# Supplementary material for: Targeting thymidine phosphorylase alleviates resistance to dendritic cell immunotherapy in colorectal cancer and promotes antitumor immunity
Source: Front Immunol. 2022 Aug 24;13:988071. doi: 10.3389/fimmu.2022.988071 (PMC9449540; doi:10.3389/fimmu.2022.988071)
Supplement: Supplementary file 2 [file DataSheet_2.pdf]

Supplementary Table 1: Reagents and Proteins			
**Protein or Reagent**	**Clone Name or Catalog Number**	**Function**	**Manufacturer**
Tipiracil Hydrochloride	S3731	Inhibitor of Thymidine phosphorylase	Selleck
Imiquimod	tlrl-imq	TLR7 Agonist	Invivogen
Recombinant mouse GMCSF	315-03	Growth factor used for DC generation	PeproTech
Recombinant mouse IL-4	214-14	Growth factor used for DC generation	PeproTech
Recombinant human GMCSF	300-03	Growth factor used for DC generation	PeproTech
Recombinant human IL-4	200-04	Growth factor used for DC generation	PeproTech
ProLong™ Gold Antifade Mountant with DAPI	P36935	DNA stain	Invitrogen
Cytoplasmic and nuclear protein extraction Kit	GX-6010AR	Isolation of cytoplasic and nuclear fraction	Genetix Biotech
Membrane protein extraction kit	89842	Isolation of membrane protein	Invitrogen

Supplementary Table 2 - Antibodies and reagents used in flow cytometry.			
**Target or Reagent**	**Clone Name or Catalog Number**	**Dilution or Working Concentration**	**Manufacturer**
Live/Dead Kit	L10119	1:200	Life Technologies
anti-CD-3	100203	2 µg/mL	Biolegend
anti-CD4	RM4-5	1:500	Biolegend
anti-CD8	53-6.7	1:500	Biolegend
anti-CD11b	M1/70	1:500	Biolegend
anti-CD11c	N418	1:500	Biolegend
anti-CD-25	PC61	1:200	Biolegend
anti-CD-40	5C3	1:20	Biolegend
anti-CD44	IM7	1:200	Biolegend
anti-CD45	A20	1:200	Biolegend
anti-CD62L	MEL-14	1:100	Biolegend
anti-CD64	X54-5/7.1	1:100	Biolegend
anti-CD69	H1.2F3	1:50	Biolegend
anti-CD80	16-10A1	1:1500	Biolegend
anti-CD86	PO3	1:100	Biolegend
anti-Granzyme B	GB11b	1:20	Biolegend
anti-CTLA4	MA5-23842	1:500	Invitrogen
anti-FOXP3	MF-14	1:100	Biolegend
anti-IFN-γ	XMG1.2	1:200	Invitrogen
anti-LAG-3	C9B7W	1:200	Biolegend
anti-mouse H2Kd	SF1-1.1	1:500	Biolegend
anti-I-A/I-E	M5/114.15.2	1:500	Biolegend
Gr-I	RB6-8C5	1:50	Biolegend
anti-NKG2D	CX5	1:500	Biolegend
anti-PD1	29F.1A12	1:200	Biolegend
anti-TIGIT	1G9	1:200	Biolegend
anti-TIM3	B8.2C12	1:100	Biolegend
anti-TNF-α	6B8	1:50	Biolegend
anti-Neuropilin-1	3725	1:500	CST
anti-TGF-β	MAB1835	1:500	R&D
anti-TYMP	PA5-116481	1:200	Invitrogen
Fc-blocker	93	1:100	Biolegend
Brilliant Violet 421™ Goat anti-rat IgG	Poly4054	1:500	Biolegend
FITC anti-rat IgG1 Antibody	MRG2a-83	1:500	Biolegend
PE anti-rat IgG1	MRG1-58	1:500	Biolegend
Alexa Fluor® 594 anti-rat IgG1	MRG1-58	1:500	Biolegend
Alexa Fluor® 647 anti-rat IgG2a	MRG2a-83	1:500	Biolegend
APC/Fire™ 750 Goat anti-rat IgG	Poly4054	1:500	Biolegend
Pacific Blue™ anti-mouse IgD	11-26c.2a	1:500	Biolegend
Alexa Fluor® 488 anti-mouse IgD	11-26c.2a	1:500	Biolegend
PE anti-mouse IgD	11-26c.2a	1:500	Biolegend
APC anti-mouse IgD	11-26c.2a	1:500	Biolegend
Brilliant Violet 711™ anti-mouse IgD	11-26c.2a	1:500	Biolegend
DyLight™ 594 Goat anti-hamster	Poly4055	1:500	Biolegend
Mouse Th1/Th2 Panel (8-plex)	741053	1:500	Biolegend

Supplementary Table 3 - ELISA Kits used		
**Target Cytokines**	**Catalouge Number**	**Manufacturer**
Mouse IFN	430804	Biolegend
Mouse IL2	DY402-05	R&D Systems
Mouse IL6	431304	Biolegend
Mouse IL10	431414	Biolegend
Mouse IL-12 (p70)	433604	Biolegend
Mouse TGF	DY1679-05	R&D Systems
Mouse TNF	430904	Biolegend
Mouse VEGF	DY493-05	R&D Systems

Supplementary Table 4 - Primary and Secondary antibodies used in IFC & IHC.			
**Antibodies**	**Clone Name or Catalog Numbe**	**Dilution**	**Manufacturer**
anti-TYMP	MA5-13542	1:100	Invitrogen
anti-Calreticulin	12238S	1:100	Cell Signaling Technology
anti-HMGB1	H9664	1:100	Sigma-Aldrich
anti-CD8	372902	1:100	Biolegend
anti-PD1	135203	1:100	Biolegend
anti-CD31	102401	1:100	Biolegend
anti-TYMP	MA5-13542	1 µg/mL	Invitrogen
anti-Ki-67	14-5698-82	2.5 µg/mL	Invitrogen
Anti-rabbit IgG (H+L), F(ab')2 Fragment (Alexa Fluor® 488 Conjugate)	4412S	1:2000	Cell Signaling Technology
Anti-rabbit IgG (H+L), F(ab')2 Fragment (Alexa Fluor® 594 Conjugate)	8889S	1:2000	Cell Signaling Technology
FITC anti-rat IgG1 Antibody	MRG2a-83	1:1000	Biolegend
Alexa Fluor® 594 anti-rat IgG1	MRG1-58	1:1000	Biolegend

Supplementary Table 5 - Primary and Secondary antibodies used in immunoblot.			
**Antibodies**	**Clone Name or Catalog Numb**	**Dilution**	**Manufacturer**
anti-Calreticulin	12238S	1:1000	Cell Signaling Technology
anti-H3	4499S	1:1000	Cell Signaling Technology
anti-HMGB1	6893S	1:1000	Cell Signaling Technology
anti-β-Actin	3700	1:1000	Cell Signaling Technology
anti-PDL1	155402	1:1000	Biolegend
E-cadherin	14472S	1:1000	Cell Signaling Technology
anti-TYMP	MA5-13542	1:1000	Invitrogen
Goat anti-Mouse IgG (H+L) HRP	31430	1:10000	Invitrogen
Goat anti-Rat IgG (H+L), HRP	31470	1:10000	Invitrogen
Goat anti-Rabbit IgG (H+L), HRP	31460	1:10000	Invitrogen

Supplementary Table 6 - Critical commercial assays Kits		
**Kits**	**Manufacturer**	**Catalouge/Code**
CellTiter 96® (MTT) Kit	Promega	G4000
CytoTox 96® Cytotoxicity Assay	Promega	G1780
Annexin V-FITC apoptosis kit	Biolegend	640914
Click-iT™ Plus TUNEL	Invitrogen	C10617
CellRox oxidative stress reagent	Molecular Probes	C10444
Novolink Polymer Detection	Leica Biosystems	RE7140-K

Supplementary Table 7 - Experimental models: Cell lines and Animals		
**Cell Line/Animal**	**Source**	**Cat. No/Identifier**
CT26	ATCC	CRL-2638
MC-38	Kerafast	ENH204-FP
HCT116	ATCC	CCL-247
HCT15	ATCC	CCL-225
CaCo2	ATCC	HTB-37
C127I	ATCC	CRL-1616
HepG2	ATCC	HB-8065
4T1	ATCC	CRL-2539
MCF7	ATCC	HTB-22
BALB/cAnNCrl	Charles River Laboratories.	http://www.hylascobio.com/index.html

Supplementary Table 8: Software and algorithms		
**Software/Packages**	**Source**	**Identifier**
FlowJo™ v10.8	BD Bioscience	https://www.flowjo.com/solutions/flowjo
OriginLab V2021b	Originlab	https://www.originlab.com/
GraphPad Prism 9	GraphPad Software	https://www.graphpad.com/
MIPAR	MIPAR Image Analysis	https://www.mipar.us/
Leica Application Suite X	Leica Microsystems	LAS_X_Core_3.7.4_23463
myImageAnalysis™	Thermo Scientific	Cat No. 62237
